# An S‐domain receptor‐like kinase regulates auxin distribution and root gravitropism via MPK signaling in Arabidopsis

**DOI:** 10.1111/tpj.71206

**Published:** 2026-11-05

**Authors:** Myint Myint San, Caoli Zhang, Yakun Peng, Meng Chen, Michelle Gallei, Lianghanxiao Sun, Mengjuan Kong, Jiří Friml, Shutang Tan

**Affiliations:** ^1^ MOE Key Laboratory for Cellular Dynamics, Center for Advanced Interdisciplinary Science and Biomedicine of IHM, Division of Molecular & Cell Biophysics, Hefei National Science Center for Physical Sciences at the Microscale, and School of Life Sciences, Division of Life Sciences and Medicine University of Science and Technology of China Hefei 230027 China; ^2^ Institute of Science and Technology Austria (ISTA) Am Campus 1 3400 Klosterneuburg Austria

**Keywords:** root system architecture, auxin, HIB1, PIN, MPK6

## Abstract

Root system architecture (RSA) varies across environments and plays essential roles in plant adaptive growth. Auxin and its polar transport have been found to be key players in the regulation of RSA. However, the molecular mechanisms underlying the regulation of RSA in fluctuating environments remain unclear. Using a forward genetic screen for root mutants under gravitropism interference conditions in the model plant *Arabidopsis thaliana*, we revealed that *HYPERSENSITIVE TO IBUPROFEN1* (*HIB1*) regulates root gravitropic growth and RSA by modulating auxin distribution in Arabidopsis. The *hib1* seedlings presented defects in primary root elongation, lateral root organogenesis, and the root gravitropic response because of abnormal auxin distribution. Notably, the gravitropic defects of *hib1* roots were correlated with increased MPK6 activity and could be rescued by loss‐of‐function *mpk6* mutation. Furthermore, cell biological results revealed that HIB1 regulated PIN protein polar distribution and abundance at the plasma membrane, indicating that HIB1 is involved in polar auxin transport and the subsequent auxin response. Indeed, genetic perturbation of *HIB1* results in impaired gravitropic root growth. Our work revealed that the HIB1‐auxin module regulates root gravitropism and RSA, providing insights into the molecular mechanisms underlying adaptive root growth.

## Significance Statement

Auxin and its polar transport are well known to regulate root gravitropism and root system architecture, and identifying the molecular framework regulating polar auxin transport and asymmetric growth is key in plant biology. Here, we report that a receptor‐like kinase, HIB1, is a novel regulator of root gravitropism and root system architecture, revealing its critical role in modulating PIN trafficking, auxin distribution, and root gravitropism.

## INTRODUCTION

The roots enable plants to anchor themselves to the ground, absorb water and nutrients from the soil, and communicate with external environmental elements. The growth status of the root system directly affects the ‘quality of life’ of plants and crop yield. However, plant roots exhibit multiple root system architectures (RSAs) or great plasticity when facing environmental diversity such that they can adapt to their surroundings or even survive (Orman‐Ligeza et al., 2013; Ötvös et al., 2021; Overvoorde et al., 2010; Roychoudhry & Kepinski, 2022; Szepesi, 2020). Root branching, which mainly consists of lateral root (LR) emergence from primary roots, root hair (RH) growth, adventitious root (AR) generation, and root length, affects RSA (Khan et al., 2016). In addition, plant roots can integrate complex environmental cues to grow directionally, including gravitropism, halotropism, and hydrotropism, which also contribute to RSA (Overvoorde et al., 2010; Roychoudhry & Kepinski, 2022). Given the importance of RSA, although the underlying mechanisms that govern RSA have been widely explored, genetic and molecular evidence that naturally regulates seedling RSA with no external pressure remains largely unknown.

Phytohormones are widely involved in modulating RSA (Jung & McCouch, 2013; Overvoorde et al., 2010). Notably, auxin acts as a multifunctional and principal endogenous signaling molecule in the regulation of plant root growth (Jung & McCouch, 2013). As one of the major regulators of LR organogenesis, the auxin‐triggered signaling network defines the LR initiation window, allows LR founder cell specificity, and promotes LR primordia development from emergence to outgrowth; within these programs, local and precise auxin concentration gradients established by polar auxin transport (PAT) play important roles via AUXIN1 (AUX1)/LIKE AUX1s (LAXs) auxin importers and PIN‐FORMED (PIN) auxin exporters (Overvoorde et al., 2010; Roychoudhry & Kepinski, 2022; Tan et al., 2021; Vanneste & Friml, 2009). In addition, multiple signaling pathways cooperate with the auxin network to modulate root growth and development, ensuring developmental plasticity under different conditions. For example, auxin can coordinate jasmonic acid (JA) homeostasis by targeting TRANSPORTER INHIBITOR1 (TIR1)/AUXIN‐SIGNALING F‐BOXs (AFBs)‐Aux/IAAs (AUXIN/INDOLE‐3‐ACETIC ACIDs) to control AR formation (Quint & Gray, 2006; Salehin et al., 2015). A relatively low level of nitrogen coordinates spatial auxin signaling to induce the function of the ROOT HAIR DEFECTIVE6 (RHD6)‐ROOT HAIRLESS‐LIKE3 (LRL3) module in RH growth (Rentel et al., 2004).

Auxin serves as a pivotal phytohormone that governs gravitropic responses in plants (Swarup et al., 2005). Auxin determines the patterning and formation of the root cap (Sabatini et al., 1999), the gravity‐sensing tissue comprising the LR cap and columella cells (CCs), and regulates the synthesis of starch granules within CCs (Zhang et al., 2019). Starch granules redistribute toward gravity following a gravistimulus; thus, LAZY/LAZY LIKE (LZY)/NEGATIVE GRAVITROPISM (NGR) proteins relocate from the statolith to the plasma membrane (PM) (Furutani et al., 2020), which is an important signal that regulates directional auxin flow. In addition, CC‐expressed PINs (PIN3 and PIN7), a major class of auxin efflux carrier, can subsequently modulate the asymmetric distribution of auxin after gravistimulation to cause the growth of roots to bend toward gravity (Blilou et al., 2005; Friml et al., 2002; Kleine‐Vehn et al., 2010), thus resulting in the dual function of auxin in the perception and transduction of gravity signals during the gravitropic response of roots. Multiple components, including kinases and phosphatases, modify the PIN protein and other auxin signaling‐related components to modulate PAT and further root gravitropism (Tan et al., 2021). For example, in a forward genetic screen investigating root growth sensitivity to the auxin transport inhibitor NPA, *EXOCYST70A3* was found to shape RSA by controlling the auxin response via interference with PIN4 distribution in LRs (Ogura et al., 2019). These studies highlight the central role of auxin, especially its distribution and signaling in shaping RSA. However, how these various environmental cues are integrated into the auxin pathway remains largely unknown.

Nonsteroidal anti‐inflammatory drugs (NSAIDs) are a group of compounds with anti‐inflammatory properties that are commonly used to alleviate and treat painful fever and inflammation in patients with conditions such as arthritis, migraine, and postoperative recovery (Lichterman, 2004; Yin et al., 1998). Since the isolation of salicylates from willow bark in the 1830s and the discovery of aspirin (acetyl salicylate) in 1897, NSAIDs have been widely applied as anti‐inflammatory drugs (Hawley et al., 2012; Lichterman, 2004; Rowlinson et al., 2003). NSAIDs function by targeting the enzyme CYCLOOXYGENASE‐2 (COX‐2), which is crucial for the biosynthesis of prostaglandins, thereby reducing inflammation and pain in the body (Duggan et al., 2011; Vane & Botting, 2003). Notably, we discovered that NSAIDs share bioactivities in plants. Some NSAIDs, such as ibuprofen (IBU), meclofenamic acid (Meclo), and flufenamic acid (Fluf), influence actin filament dynamics by targeting the immunophilin‐like protein TWISTED DWARF1 (TWD1) to modulate the dynamics of actin filaments and endomembrane trafficking, thus impairing PIN2 protein trafficking and root gravitropic growth (Tan et al., 2020). The synthetic NSAID naproxen directly binds to PIN proteins, competitively inhibiting PIN‐mediated auxin transport and thereby triggering severe root growth defects during gravitropism (Xia et al., 2023). Triple inhibition describes a suite of growth defects tightly linked to auxin function, including inhibited primary root elongation, suppressed LR initiation, and defective root gravitropism (Fendrych et al., 2018; Tan et al., 2020; Wang et al., 2005, 2015). Moreover, exogenous NSAIDs drastically repress primary root growth and LR initiation, resulting in a typical triple inhibition effect. Nevertheless, the molecular regulators that control RSA mediated by NSAIDs remain poorly characterized.

In this study, to identify novel regulators of root gravitropism and RSA, we used ethyl methanesulfonate (EMS) mutagenesis in the model plant *Arabidopsis thaliana* followed by IBU treatment and identified *HYPERSENSITIVE TO IBUPROFEN1* (*HIB1, AT4G32300*, previously annotated *S DOMAIN‐2 5, SD2‐5*), a gene that can contribute to the regulation of RSA in Arabidopsis seedlings by controlling PAT. Compared with the WT seedlings, the *hib1* mutation resulted in shortened primary roots, reduced LRs, and decreased root gravitropism, which are classical defects of RSA. Further study revealed that *hib1* interfered with *PIN* (*PIN1*, *PIN2*, *PIN3*, and *PIN7*) expression patterns and polar distribution, suggesting that HIB1, as an independent module, specifically regulates the PIN‐related auxin pathway.

## RESULTS

### *HIB1* is involved in modulating root gravitropism and RSA in Arabidopsis

To explore the genetic and molecular mechanisms regulating plant root gravitropism and RSA, we performed a forward genetic screen with an EMS population in the Arabidopsis *PIN2::PIN2‐GFP* background. In the screen, the M_2_ plants were treated with 40 μM IBU, and then we identified *hypersensitive to ibuprofen* (*hib*) and *resistant to ibuprofen* (*rib*) mutants on the basis of their sensitivity to the gravitropism‐interfering compound IBU (Tan et al., 2020). In this study, we focused on *hib1‐1*, which presented a hypersensitive response to IBU treatment and defects in root length, gravitropic response, and LR growth (Figure 1A–D). Phenotypic analysis revealed that *hib1‐1* presented slightly short primary roots on normal Murashige and Skoog (MS) media and exhibited increased sensitivity to IBU in terms of root elongation (Figure 1A,B), LR formation (Figure 1C), and root gravitropism (Figure 1D). Further pharmacological treatments with the auxin transport inhibitors naproxen and NPA revealed a similar hypersensitivity response for *hib1‐1* (Figure S1A–D). These results suggest that the corresponding gene for the *hib1‐1* mutant might encode an essential component involved in regulating the auxin pathway and thus root development.

**Figure 1:**
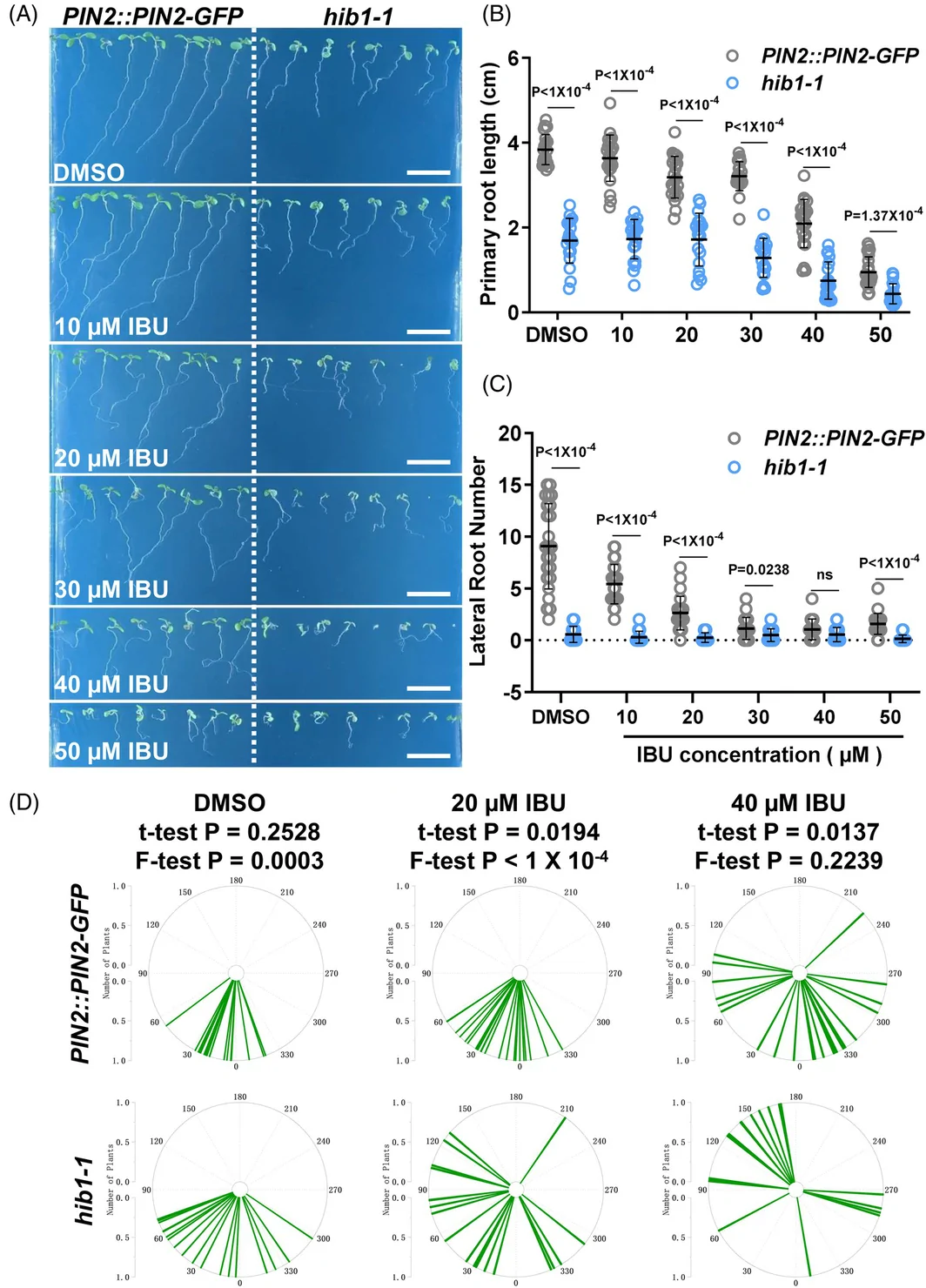
The physiological effects of ibuprofen (IBU) on the growth and development of *PIN2::PIN2‐GFP* and *hib1‐1* roots. (A) Representative images showing the morphological changes in the roots of 7‐day‐old ethyl methanesulfonate (EMS) mutants *hib1‐1* (in the *PIN2::PIN2‐GFP* background) and *PIN2::PIN2‐GFP* treated with different concentrations of IBU. The seedlings were subsequently grown on Murashige and Skoog (MS) media supplemented with various concentrations of IBU. DMSO was used as the solvent control. Scale bars, 1 cm. (B, C) Quantitative analysis of primary root length (B) and lateral root number (C) in 7‐day‐old *hib1‐1* and *PIN2::PIN2‐GFP* plants treated with IBU. IBU treatment significantly inhibited primary root elongation and lateral root formation in 7‐day‐old *PIN2::PIN2‐GFP* and *hib1‐1* seedlings and impaired root gravitropism. The number of lateral roots in 7‐day‐old *PIN2::PIN2‐GFP* and *hib1‐1* seedlings treated with IBU was quantified. *n* = 21, 23, 24, 22, 23, and 24 replicates for PIN2::PIN2‐GFP; *n* = 19, 21, 18, 19, 19, and 20 replicates for hib1‐1 (B); *n* = 24, 24, 24, 23, 20, and 24 replicates for PIN2::PIN2‐GFP; and *n* = 19, 20, 19, 20, 20, and 21 replicates for hib1‐1 (C). The data are presented as the means ± SDs, and the dots represent individual values. *P* values were determined via an unpaired *t*‐test with Welch's correction for the indicated *PIN2::PIN2‐GFP* and *hib1‐1* pairs at a specific concentration of IBU. (D) Root gravitropic response analysis of 7‐day‐old *PIN2::PIN2‐GFP* and *hib1‐1* seedlings treated with IBU. IBU treatment affected root gravitropism. The polar bar charts show the root tip angles of individual seedlings, which were used to quantify the extent of gravitropic disruption. *n* = 20, 23, and 23 for *PIN2::PIN2‐GFP*, and *n* = 20, 19, and 19 for *hib1‐1*, respectively. Each line represents the root tip angle of an individual seedling in the polar bar charts. Unpaired *t*‐tests were used to assess significant differences among means, and *F* tests were employed to evaluate differences in variance. DMSO served as the control.

We generated a plant population by crossing *hib1‐1* (in the *PIN2::PIN2‐GFP* background) and *PIN2::PIN2‐GFP*. The F_1_ plants exhibited a parental *PIN2::PIN2‐GFP* root phenotype (Figure S2A–C), suggesting a recessive mutation for the *hib1‐1*. We subsequently performed next‐generation sequencing (NGS) with a bulk F_2_ backcross population to identify candidate genes involved in the regulation of *hib1‐1* root growth in Arabidopsis. The results revealed that the *hib1‐1* line harbored a G‐to‐A point mutation at position 196 of the *HYPERSENSITIVE TO IBUPROFEN1* (*HIB1, AT4G32300*, previously annotated *S DOMAIN‐2 5, SD2‐5*) coding region, leading to an amino acid substitution from valine to isoleucine (V66I) (Figure S3A,B). *HIB1* encodes a receptor‐like kinase (RLK) that comprises a highly conserved serine/threonine protein kinase catalytic domain and an extracellular domain composed of a G‐type lectin region and an S‐locus region. To verify the biological functions of *HIB1*, we obtained a T‐DNA insertional mutant (SALK_076637), which we named *hib1‐2* (Figure S3B–D). We conducted polymerase chain reaction (PCR) to verify the presence of T‐DNA insertions in *hib1‐2* (Figure S3C), and real‐time PCR results revealed that *HIB1* was not expressed in *hib1‐2* (Figure S3D). Phenotypic analysis revealed that *hib1‐2* presented slightly short primary roots on normal MS media and exhibited increased sensitivity to IBU in terms of root elongation (Figure S4A,B), LR formation (Figure S4C), and root gravitropism (Figure S4D), which was similar to that of *hib1‐1*. Moreover, the *hib1‐2* mutant also displayed hypersensitivity to the auxin transport inhibitors NPA and naproxen (Figure S5A–D). To further confirm the validity of *HIB1* as a candidate gene, we conducted an allelic test by crossing *hib1‐1* and *hib1‐2* (Figure S6). Results showed that the F_1_ plants presented severely restricted primary root elongation (Figure S6A,B), a defective gravitropic response in roots (Figure S6C), and a hypersensitive response to IBU treatment (Figure S6A–C), with phenotypic traits similar to those observed in both the *hib1‐1* and *hib1‐2* mutants. To further investigate whether *HIB1* is the true causal gene for the corresponding root phenotype, we tested whether *HIB1* can rescue the impaired root development of *hib1‐1*. Therefore, the *pHIB1::HIB1‐mCherry* construct was generated and introduced into the *hib1‐1* background. The resulting transgenic lines fully rescued abnormal root growth and overall development, resembling that of the *PIN2::PIN2‐GFP* seedlings (Figure S7A–C). These data suggest that *HIB1* is the candidate gene responsible for root growth defects in the *hib1‐1* mutant, indicating the potential involvement of *HIB1* in the regulation of RSA by the auxin pathway.

### Localization and functional characterization of HIB1

Next, we observed the expression pattern and subcellular localization of *HIB1* in Arabidopsis. To investigate the subcellular localization of HIB1 in roots, HIB1‐mCherry was expressed in *hib1‐1* and *hib1‐2* under its native promoter (*pHIB1::HIB1‐mCherry;hib1‐1*, *pHIB1::HIB1‐mCherry;hib1‐2*; Figures S7 and S8), HIB1‐GFP was expressed in the WT under a CaMV 35S promoter (*35S::HIB1‐GFP* in the WT; Figure S9), and *pHIB1::GUS* was expressed in the WT (Figure 2). HIB1 predominantly accumulated in the root cap and epidermis in *pHIB1::HIB1‐mCherry;hib1‐1* and *pHIB1::HIB1‐mCherry;hib1‐2* (Figures S7C and S8E). With GUS staining, we observed that *HIB1* was also expressed in the root tip, the LR at different developmental stages, and the hypocotyl, especially at the junction region between the primary root and the hypocotyl, as well as in leaf tissues, particularly in guard cells and leaf mesophyll cells (Figure 2A). Nevertheless, the endogenous expression level of *HIB1* is relatively low in plant roots, resulting in weak HIB1‐mCherry fluorescence under normal growth conditions. Notably, β‐estradiol induction markedly increased *HIB1* expression in roots, which enables clear visualization of its tissue‐specific expression pattern (Figures S7C and S8E). The visible fluorescence signals confirm the validity of the complementary lines, while such induced expression disrupts the native subcellular localization of HIB1. Instead, in *35S::HIB1‐GFP*, imaging via confocal laser scanning microscopy (CLSM) revealed that HIB1‐GFP predominantly localized to the PM, where it colocalized with the signal of the lipophilic fluorescent dye FM4‐64 (Figure 2B,C; Figure S9C,D).

**Figure 2:**
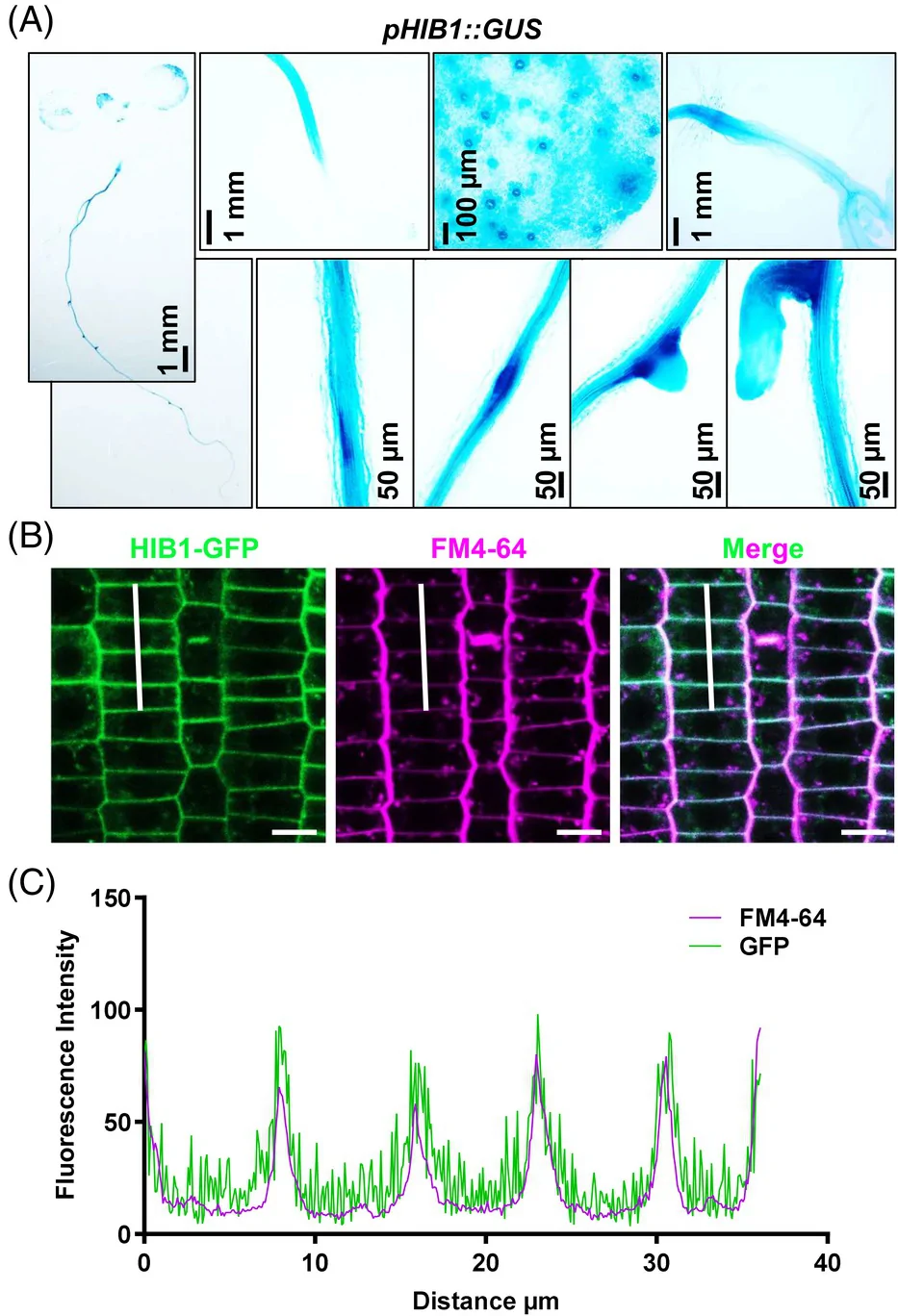
Tissue and subcellular expression patterns of HIB1. (A) GUS staining of transgenic plants expressing *pHIB1::GUS*. Scale bars are indicated on each panel. (B) Representative images showing the subcellular localization of HIB1. HIB1‐GFP was expressed in Arabidopsis driven by the CaMV 35S promoter. FM4‐64 staining was used as a marker for plasma membrane (PM). Scale bar, 10 μm. (C) Colocalization analysis of fluorescence intensity for 35S::HIB1‐GFP and FM4‐64 staining signals at the positions indicated by the white lines in (B).

To elucidate the biological functions of *HIB1* in RSA, we analyzed the phenotype of the EMS mutagenesis mutant *hib1‐1* and the T‐DNA insertional mutant *hib1‐2* at developmental stages competent for LR formation. We found that root morphological characteristics were markedly altered in *hib1‐1* and *hib1‐2*. Our observations revealed that the LR of *hib1‐2* has significantly disordered growth angles (Figure 3A,C), resembling those of the *hib1‐1* mutant (Figure 3B,F). Moreover, older *hib1‐2* and *hib1‐1* seedlings presented markedly reduced LR numbers and shorter primary root lengths, which contributed to RSA establishment (Figure 3A,B,D,E,G,H). Overall, compared with the WT plants, the *hib1‐2* plants presented a specific ‘triple inhibition’ phenotype, and these growth defects were further exacerbated when the plants were treated with IBU (Figure 1; Figure S4), which involved gravitropism defects, shorter primary roots and less LR formation. As expected, the *pHIB1::HIB1‐mCherry;hib1‐2* transgenic line could also rescue the developmental defects in *hib1‐2* (Figure S8A,B,E). However, the size of the root apical meristem was not obviously altered in *hib1‐2* (Figure S8C,D). Interestingly, while *HIB1* overexpression (*HIB1‐OX*) lines did not change under normal conditions, these plants were hypersensitive to IBU treatment (Figure S9A,B,E). These findings suggest that both the deficiency and overaccumulation of HIB1 impair a regulatory pathway governing RSA.

**Figure 3:**
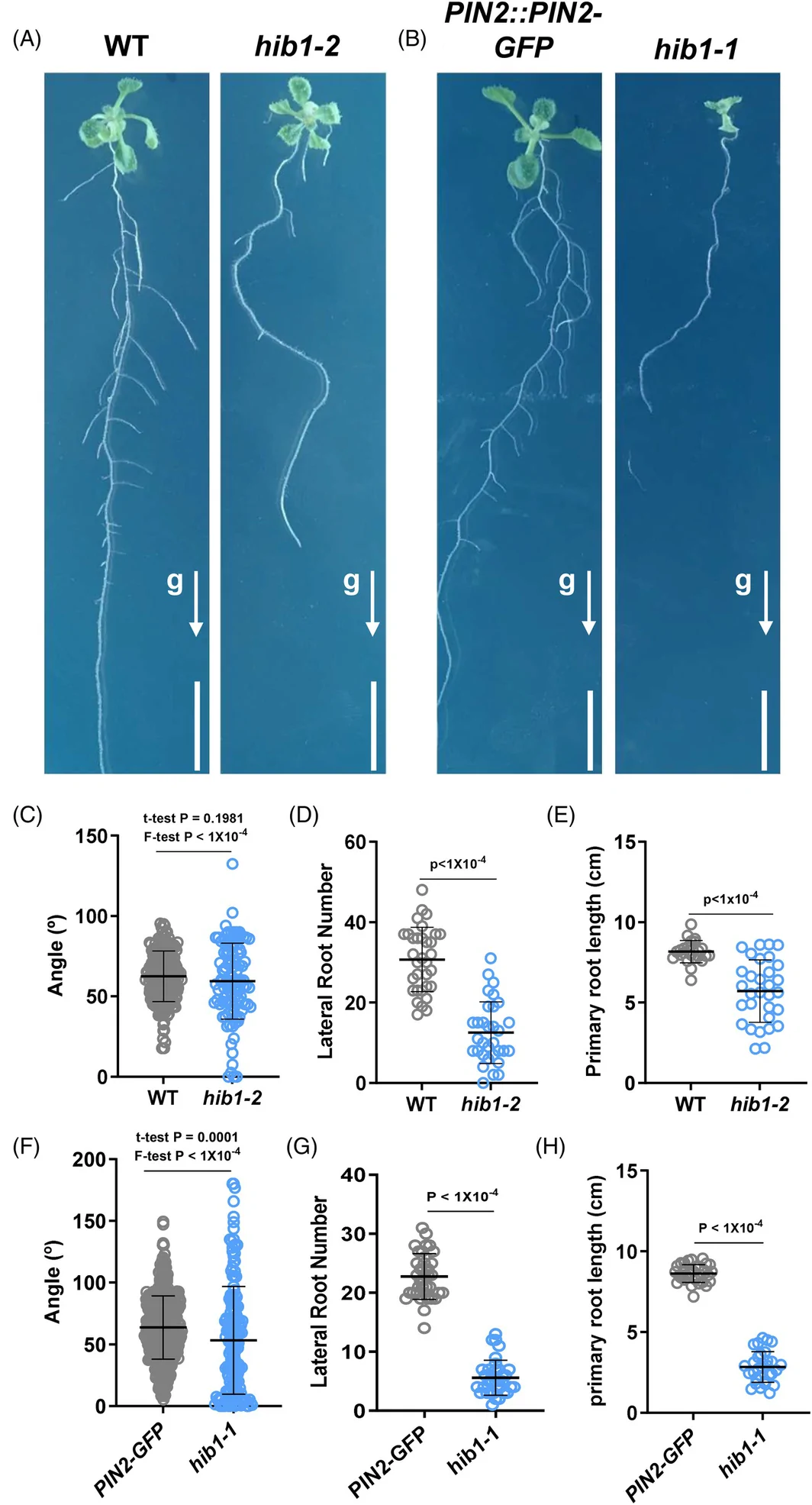
*hib1‐2* exhibited severe lateral root growth defects. (A, B) Representative images showing that the gravitropic setpoint angle (GSA) of lateral roots was impaired in *hib1‐2* seedlings (A) and *hib1‐1* seedlings (B). WT and *hib1‐2* seedlings (A) and *PIN2::PIN2‐GFP* and *hib1‐1* (B) seedlings were grown on Murashige and Skoog (MS) media for 13 days, after which representative images were captured. The arrow marked with ‘*g*’ represents the direction of gravity. Scale bars, 1 cm. (C, F) Distribution of lateral root angles in WT and *hib1‐2* seedlings (C) and *PIN2::PIN2‐GFP* and *hib1‐1* seedlings (F). Lateral root angles relative to the plumb line (gravity vector) were measured at a spot 2 mm away from the base of the lateral root. *n* = 496 for WT and *n* = 255 for *hib1‐2* (B), *n* = 561 for *PIN2::PIN2‐GFP* and *n* = 168 for *hib1‐1* (F). The data are presented as the means ± SDs, and the dots represent individual values. Unpaired *t*‐tests were used to assess significant differences among means, and *F* tests were employed to evaluate differences in variance. (D, G) Quantitative analysis of the number of lateral roots in 13‐day‐old WT and *hib1‐2* seedlings (D) and *PIN2::PIN2‐GFP* and *hib1‐1* seedlings (G). *n* = 33 for WT and *n* = 32 for *hib1‐2* (D), *n* = 36 for *PIN2::PIN2‐GFP* and *n* = 35 for *hib1‐1* (G). The data are presented as the means ± SDs, and the dots represent individual values. (E, H) Quantitative analysis of primary root length in 13‐day‐old WT and *hib1‐2* seedlings (E) and *PIN2::PIN2‐GFP* and *hib1‐1* seedlings (H). *n* = 24 for WT and *n* = 31 for *hib1‐2* (E), *n* = 32 for PIN2::PIN2‐GFP and *n* = 31 for *hib1‐1* (H). The data are presented as the means ± SDs, and the dots represent individual values. *P* values were determined by comparing WT and *hib1‐2* or *PIN2::PIN2‐GFP* and *hib1‐1* via an unpaired *t*‐test with Welch's correction.

Auxin plays a central regulatory role in RSA, especially in root gravitropism (Jung & McCouch, 2013; Overvoorde et al., 2010; Swarup et al., 2005). We tested the root gravitropic response of the *hib1‐2* mutant. WT and *hib1‐2* seedlings were subjected to gravistimulation via 90° reorientation. Compared with the WT roots, the root tip in *hib1‐2* bent down toward gravity more slowly (Figure 4A,B), and this delayed gravitropic response was independent of the slower primary root growth observed in *hib1‐2* (Figure 4C).

**Figure 4:**
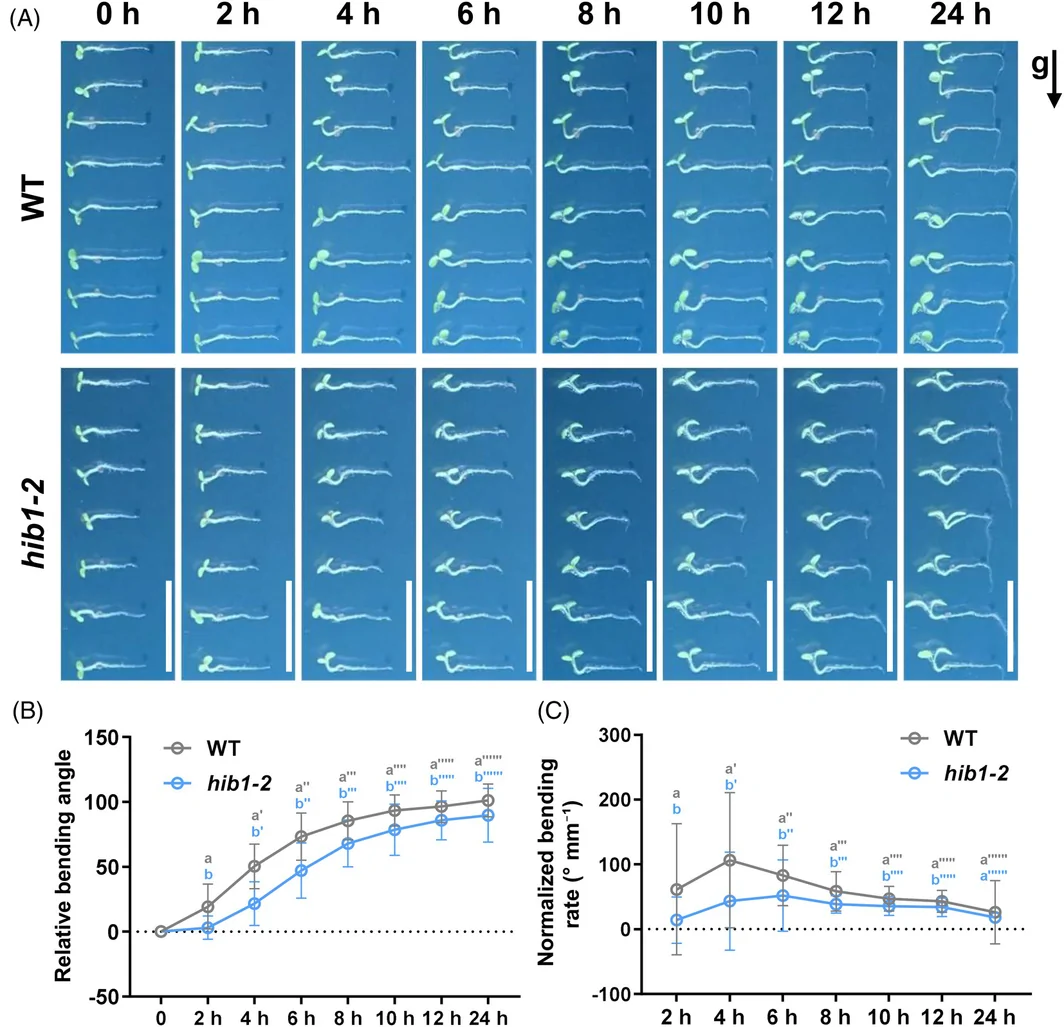
Loss of function of *HIB1* leads to an impaired root gravitropic response in the T‐DNA mutant *hib1‐2*. (A) Representative images showing that the gravitropic response was impaired in the *hib1‐2* mutant. Five‐day‐old WT and *hib1‐2* seedlings were grown on Murashige and Skoog (MS) media and reoriented at 90° for an additional 24 h, and representative images were captured at the specified time points postreorientation indicated in the diagram to document the gravitropic response. Scale bars, 1 cm. (B) Quantitative analysis of the root tip angles of WT and *hib1‐2* seedlings from 0 to 24 h following 90° reorientation. The root tip bending kinetics were assessed to determine the speed of the gravitropic response. *n* = 63 for WT and *n* = 60 for *hib1‐2*. (C) Time‐course analysis of normalized root gravitropic bending efficiency in WT and *hib1‐2* mutant seedlings. Root gravitropic bending efficiency was quantified at the indicated time points after 90° gravistimulation. Normalized bending efficiency is expressed as the bending angle per unit root length (° mm^−1^). The data are presented as the means ± SDs. Different letters indicate significant differences according to an unpaired *t*‐test with Welch's correction.

Taken together, these findings suggest that *HIB1* participates in the regulation of RSA, likely by regulating the auxin pathway, which is likely involved in the auxin response and PAT.

### *hib1‐2* interferes with the auxin response and auxin (re)distribution during the root gravitropic response

The above ‘triple inhibition’ phenotype has been reported to be closely associated with auxin. To assess whether *hib1‐2* interferes with the auxin response, we used the auxin‐responsive fluorescent reporter line *DR5rev::GFP* (Friml et al., 2003) to study the cellular auxin response in WT and *hib1‐2*. In the WT, the *DR5* signal was expressed in the central column from quiescent center (QC) cells to the columella root cap tip (Figure 5A). However, in *hib1‐2*, the region in which DR5 was expressed decreased, and the DR5 signal intensity further decreased significantly (Figure 5A,B; Figure S10). Additionally, exogenous IAA application did not trigger an increase in DR5 signal intensity in *hib1‐2* (Figure S10C,D). We next tested the response to exogenous IAA treatment in WT and *hib1‐2* plants. Exogenous IAA also failed to rescue the root gravitropic defects in *hib1‐2*, whereas higher concentrations of IAA inhibited primary root elongation (Figure S11A–C). Furthermore, although IBA, another endogenous auxin, triggered LR emergence in *hib1‐2* and *hib1‐1*, this inductive effect was weakened (Figure S12A–D). Taken together, these observations confirm that *hib1* is involved in impaired auxin responses.

**Figure 5:**
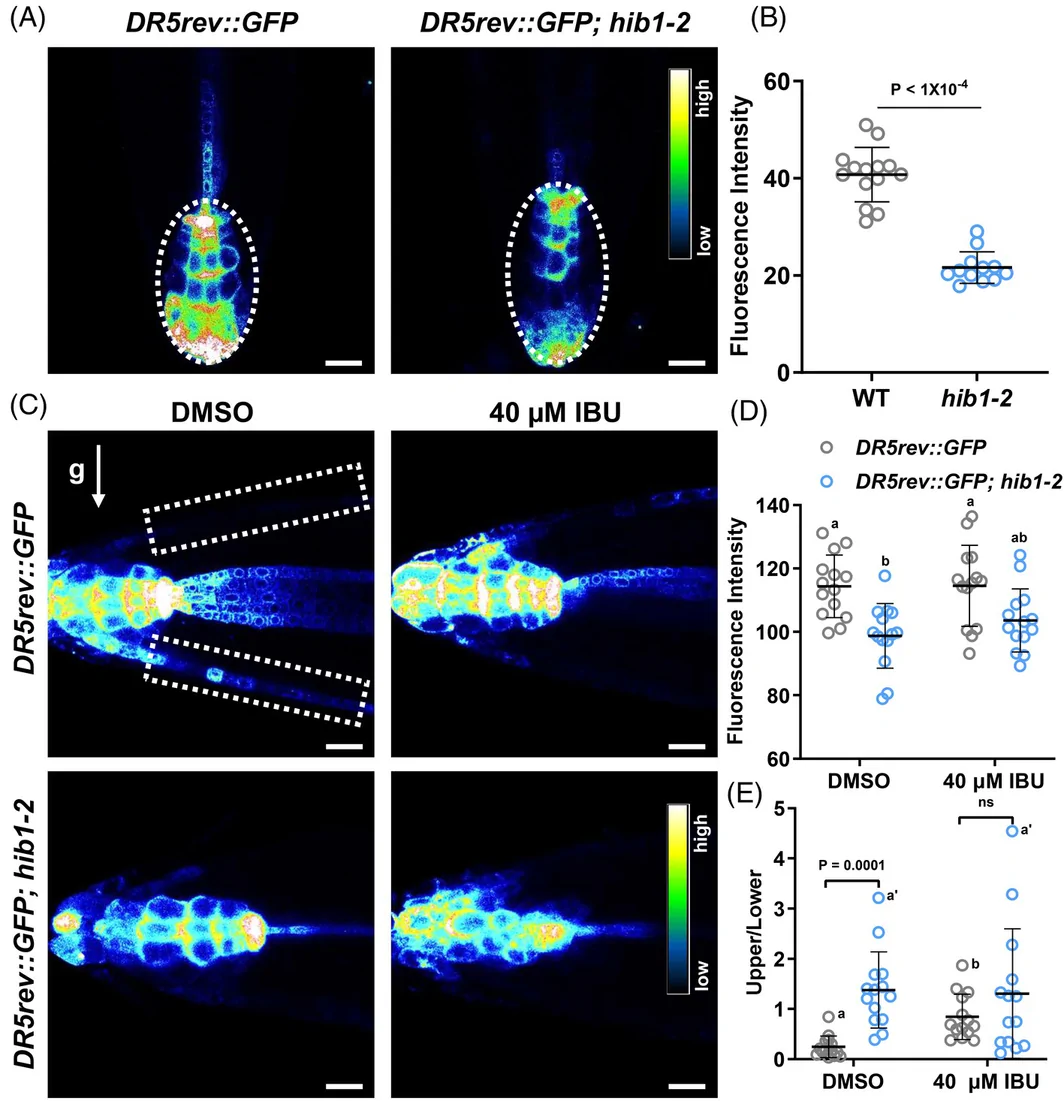
Defects in gravi‐stimulated DR5rev::GFP relocation in the *hib1‐2* mutant. (A) Representative images showing that the fluorescence intensity of DR5rev::GFP was weakened in *hib1‐2* cells. Five‐day‐old *DR5rev::GFP* and *DR5rev::GFP;hib1‐2* seedling root tips were imaged via confocal laser scanning microscopy (CLSM) in the GFP channel. Scale bars, 20 μm. The dotted white circles indicate the regions quantified. (B) Quantitative analysis of GFP fluorescence intensity in *DR5rev::GFP* and *DR5rev::GFP;hib1‐2* root tips shown in Figure 5A. *n* = 14 replicates for *DR5rev::GFP* and *n* = 12 replicates for *DR5rev::GFP;hib1‐2*. (C) Representative images showing that the DR5rev::GFP redistribution pattern under 90° reorientation was suppressed in *hib1‐2*. Five‐day‐old *DR5rev::GFP* and *DR5rev::GFP;hib1‐2* seedlings grown on Murashige and Skoog (MS) media were transferred to DMSO/ibuprofen (IBU)‐containing plates and rotated 90° for an additional 4 h. Scale bars, 20 μm. The dotted white rectangles indicate the regions quantified. The arrow marked with ‘*g*’ indicates the direction of gravity. (D) Quantitative analysis of GFP fluorescence intensity in *DR5rev::GFP* and *DR5rev::GFP;hib1‐2* root tips shown in Figure 5C. *n* = 14 replicates for *DR5rev::GFP* and *DR5rev::GFP;hib1‐2*. (E) The ratio of GFP fluorescence intensity in the upper to lower root tips was measured to indicate DR5 signal relocation in *DR5rev::GFP* and *DR5rev::GFP;hib1‐2*. *n* = 14 replicates for *DR5rev::GFP* and *DR5rev::GFP;hib1‐2*. The data are presented as the means ± SDs, and the dots represent individual values. *P* values were calculated by comparing two different plant lines via an unpaired *t*‐test with Welch's correction. Different letters represent significant differences; *P* < 0.05; one‐way anova with Tukey's multiple comparison test (D). Different letters indicate significant differences according to an unpaired *t*‐test with Welch's correction (E).

Is PAT affected in *hib1‐2*? Next, following a gravistimulus for *DR5rev::GFP* and *DR5rev::GFP;hib1‐2* by 90° reorientation, we observed a significantly impaired response to gravistimulation in the roots of *DR5rev::GFP;hib1‐2*. Confocal images revealed an increase in the lower lateral root cap under gravistimulation by 90° reorientation in *DR5rev::GFP* (Figure 5C,E). However, this gravistimulation‐induced asymmetric DR5 accumulation on the lower root side was absent in *hib1‐2* (Figure 5C,E), suggesting a defect in asymmetric auxin distribution in *hib1‐2*. Whereas exogenous IBU significantly suppressed DR5 signal redistribution in the WT, no further impairment of DR5 signal redistribution was observed in *hib1‐2*, where this process was already intrinsically impaired (Figure 5C,E). Furthermore, the GFP signal in the root tip of *hib1‐2* was also drastically attenuated relative to WT following gravistimulation (Figure 5C,D). These results indicate that the defect in the gravitropic response might be due to impaired auxin transport in *hib1‐2* during root gravitropism. This defect in auxin transport also explains the hypersensitivity of the *hib1* mutant to auxin transport inhibitors.

In addition, upon treatment with 20 μM IBU (a moderate concentration), the DR5 signal intensity in *hib1‐2* cells recovered to levels comparable to those in untreated WT cells, and the DR5 expression domain was expanded (Figure S10A,B). Increasing the IBU concentration to 40 μM (high concentration) further reduced the DR5 signal intensity in *hib1‐2*. Although WT and *hib1‐2* displayed comparable trends in DR5 signaling upon IBU treatment, high‐concentration IBU fully restored the DR5 signal intensity in *hib1‐2* to its untreated basal level. In the WT, high‐dose IBU reduced the DR5 signal, yet no significant difference was detected between the 20 and 40 μM IBU treatments (Figure S10A,B). Given the disturbance of PIN2 by IBU (Tan et al., 2020) and root agravitropism in *hib1‐2*, we speculate that, owing to intrinsic defects in auxin transport in *hib1‐2*, auxin trapping triggered by high‐concentration IBU might accelerate IAA inactivation in *hib1‐2*. Combined with the impaired auxin response, these interferences cause the DR5 signal intensity to rapidly decrease back to the basal level of untreated *hib1‐2*, a phenomenon not observed in the WT. These observations further demonstrate that *hib1‐2* is hypersensitive to IBU and imply the presence of defective auxin transport in *hib1‐2*.

### *HIB1* deficiency impairs the endosomal trafficking of PIN2

PAT is essential for maintaining the precise distribution of auxin, which further depends on the polar subcellular localization of PINs, a class of auxin efflux carriers (Tan et al., 2021). Notably, to maintain polar localization, PINs cycle between the PM polar domains and endosomal compartments in a continuous and dynamic manner instead of ‘sitting’ statically at the PM (Tan et al., 2021). To date, several components that participate in the delivery of PINs to the PM have been reported. ADP‐ribosylation factor (ARF) small GTPases regulate vesicle trafficking by coordinating coat protein activity, phospholipid metabolism, and actin structure at the membrane surface, whereas guanine nucleotide‐exchange factors (GEFs) are ARF activators that catalyze the exchange of GDP for triphosphate nucleotides (Casanova, 2007; Geldner et al., 2003; Kleine‐Vehn et al., 2009; Naramoto et al., 2010, 2014). Brefeldin A (BFA), a small‐molecule inhibitor, targets multiple BFA‐sensitive ARF‐GEF proteins, thus perturbing endomembrane trafficking across distinct Golgi subdomains and the trans‐Golgi network (TGN)/early endosome and subsequently blocking the recycling of cargo proteins such as PIN auxin transporters (Chardin & McCormick, 1999; Geldner et al., 2001; Richter et al., 2014; Steinmann et al., 1999). BFA treatment disrupted the proper polar localization of the PINs by blocking recycling. In addition, BFA also acts as an inhibitor of *de novo*‐synthesized PIN2 secretion and endocytosis during polarity re‐establishment. BFA is a great tool for revealing and visualizing secretion, endosomal trafficking, and recycling.

Previous reports have shown that PIN auxin transporters are essential for PAT to regulate the root gravitropic response. To investigate whether there are some differences in the cellular polar distribution of PIN2 between the *PIN2::PIN2‐GFP* fluorescent reporter line and *PIN2::PIN2‐GFP;hib1‐2*, we imaged the changes in the expression pattern of *PIN2* in WT and *hib1‐2* (Figure 6; Figure S14) and observed that, compared with those in WT, the overall fluorescence intensity of PIN2‐GFP and the distribution of PIN2‐GFP toward the apical membrane of the root cells in *hib1‐2* were significantly lower (Figure 6A–C; Figure S14C). These findings demonstrate that the loss of *HIB1* affects both PIN2 protein abundance and the maintenance of PIN2 polar distribution in roots. Among the eight members of the Arabidopsis PIN family, five PIN proteins widely participate in the regulation of root growth (Billou et al., 2005; Krecek et al., 2009). We therefore also analyzed the changes in the fluorescence of PIN1::PIN1‐GFP, PIN3::PIN3‐GFP, and PIN7::PIN7‐GFP in WT and *hib1‐2* and detected significant decreases in the GFP signal and expression pattern changes in *hib1‐2* (Figure S13A–F). The *hib1‐2* mutation led to a decrease of PIN3 and PIN7 in the stele and the root cap as well as a decrease of PIN1 in the stele (Figure S13A–F), suggesting that *hib1‐2* indeed impaired *PIN* expression and patterning. Since maintaining PIN2 polarity requires transcytosis and recycling, we treated *PIN2::PIN2‐GFP* and *PIN2::PIN2‐GFP;hib1‐2* with BFA. In the WT, BFA treatment caused the internalization of the PIN2 protein in a large stack of endomembrane compartments (Figure 6D,E). However, the internalization of the PIN2 protein in the endomembrane compartments was significantly decreased in *hib1‐2* (Figure 6D,E). Notably, although IBU treatment markedly suppressed PIN2 recycling in the WT, it did not further influence PIN2 recycling in *hib1‐2* (Figure S14A,B). This observation stems primarily from pre‐existing defects in PIN2 recycling in *hib1‐2*.

**Figure 6:**
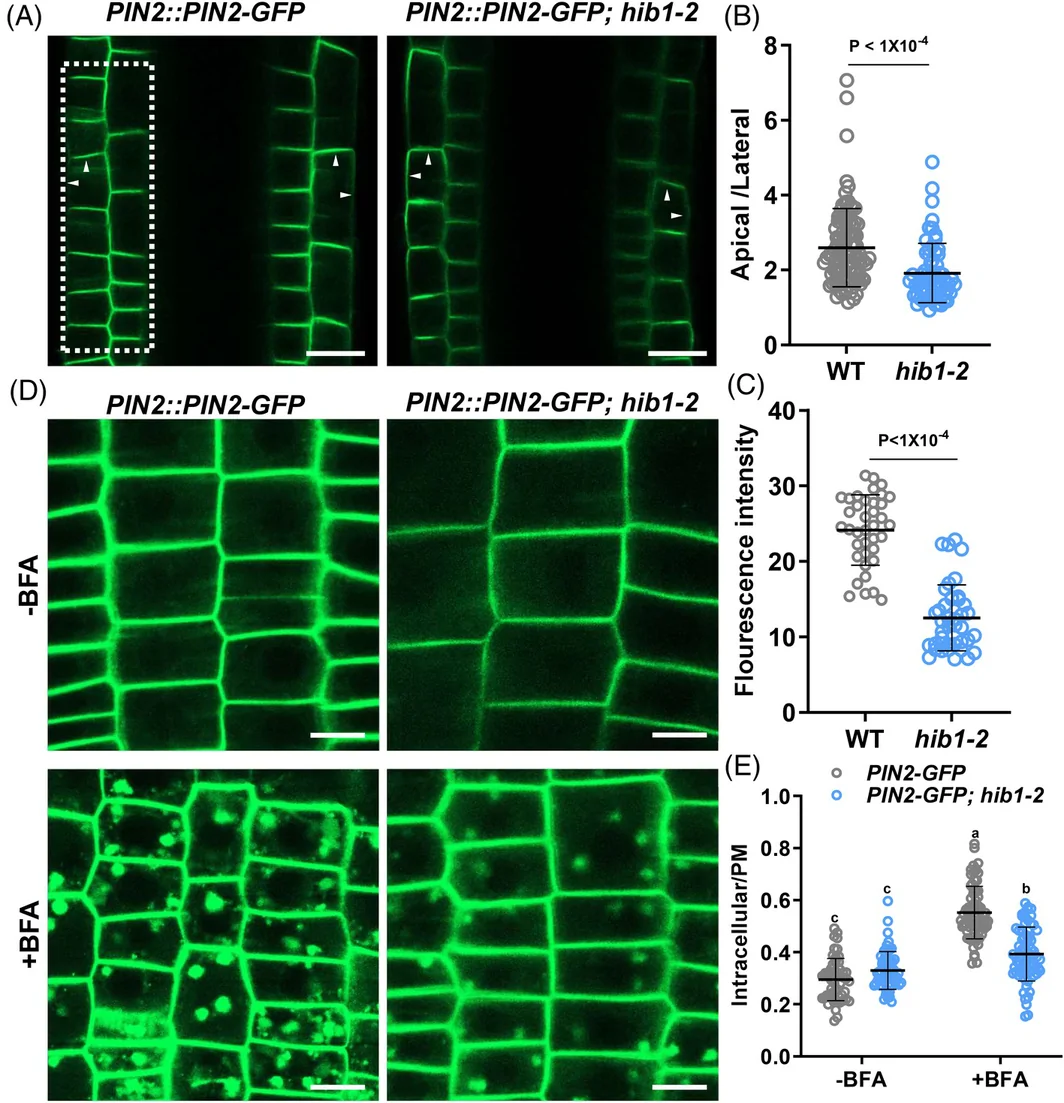
*hib1‐2* interferes with PIN2 protein abundance, polarity, and trafficking. (A) Representative images showing that loss of function of *HIB1* impaired the subcellular distribution and abundance of the PIN2 protein. Five‐day‐old seedlings were grown continuously on Murashige and Skoog (MS) media, and the signal was imaged via confocal laser scanning microscopy (CLSM) in the GFP channel. The arrows indicate the apical and lateral plasma membrane (PM) analyzed in epidermal cells. Scale bar, 20 μm. (B, C) Analysis of changes in the polar localization and abundance of PIN2‐GFP in the epidermal and cortical cells of WT and *hib1‐2 plants*. The ratio of PIN2‐GFP fluorescence intensity between the apical and lateral PM (B) and the distribution of PIN2‐GFP fluorescence intensity (C) in epidermal and cortical cells were measured with ImageJ software. *n* = 94 for *PIN2::PIN2‐GFP* and *n* = 63 for *PIN2::PIN2‐GFP;hib1‐2* in (B). *n* = 38 for *PIN2::PIN2‐GFP* and *n* = 40 for *PIN2::PIN2‐GFP;hib1‐2* in (C). (D) Confocal images showing the localization of *PIN2::PIN2‐GFP* in WT and *hib1‐2 cells* treated with 50 μM BFA for 30 min or not. Five‐day‐old WT and *hib1‐2* seedlings were grown continuously on MS media and then treated with 50 μM BFA for an additional 30 min or not. The roots were imaged via CLSM in the GFP channel. Scale bars, 10 μm. (E) The ratio of PIN2‐GFP fluorescence intensity of the intracellular to that of the PM was measured in WT and *hib1‐2*. *n* = 55 for *PIN2::PIN2‐GFP*, −BFA, *n* = 75 for *PIN2::PIN2‐GFP*, +BFA, *n* = 60 for *PIN2::PIN2‐GFP;hib1‐2*, −BFA, *n* = 70 for *PIN2::PIN2‐GFP;hib1‐2*, +BFA. The data are presented as the means ± SDs, and the dots represent individual values. *P* values were calculated by comparing two different plant lines via an unpaired *t*‐test with Welch's correction. Different letters represent significant differences; *P* < 0.05; one‐way anova with Tukey's multiple comparison test (E).

We also performed FM4‐64 uptake assays to monitor the bulk endocytosis in WT and *hib1‐2* roots. Quantitative analysis revealed that FM4‐64 internalization is significantly reduced in *hib1‐2*, indicating that general endocytosis is severely impaired in *hib1‐2* (Figure S14D,E), which most likely compromises PIN2 endocytosis mediated by clathrin (Du et al., 2013; Tan et al., 2020). These results also strongly suggest that the effect of *HIB1* loss of function on endomembrane trafficking is not restricted to PIN proteins but rather reflects a broader defect in PM protein dynamics. These data suggest that the loss of *HIB1* interferes with the establishment of polar localization and endosomal trafficking of the PIN protein, thus influencing PAT. Impaired PAT consequently disturbs the distribution of auxin, ultimately leading to defects in root gravitropic responses.

### HIB1 regulates root gravitropic growth via MPK6 signaling

We hypothesize that HIB1 may act as a PM‐localized RLK to perceive extracellular signaling and then transduce this signal to intracellular components. Previous reports suggest that the RLK signaling system usually involves PM‐associated RLK pairs and RLCK components, as well as the downstream MPK kinase cascade (Bi & Zhou, 2017; Dievart et al., 2020; Shiu & Bleecker, 2003). To test whether HIB1 functions via the MPK signaling pathway, we investigated MPK activity. Western blot analysis revealed increased MPK6 and MPK3 activity in *hib1‐2* and *hib1‐1* compared with WT and *PIN2::PIN2‐GFP*, respectively, which was further confirmed by an *hib1‐2 mpk6‐2* genetic cross (Figure 7A; Figure S15A). Notably, the root growth and gravitropic defects of *hib1‐2* were reversed by the *mpk6‐2* mutation (Figure 7B,C). However, the loss of *MPK3* did not rescue the growth defects in *hib1‐2* (Figure S15B,C), suggesting that MPK6 plays a dominant role in HIB1‐mediated root growth. Previous studies have shown that MPK6 can phosphorylate the cytoplasmic loops of PIN proteins (PIN‐HLs) (Wang et al., 2024), which leads to a decrease in PIN proteins at the PM, which is in line with our observation of the *hib1‐2* mutant. These findings suggest that HIB1 regulates root gravitropic growth via MPK6 signaling.

**Figure 7:**
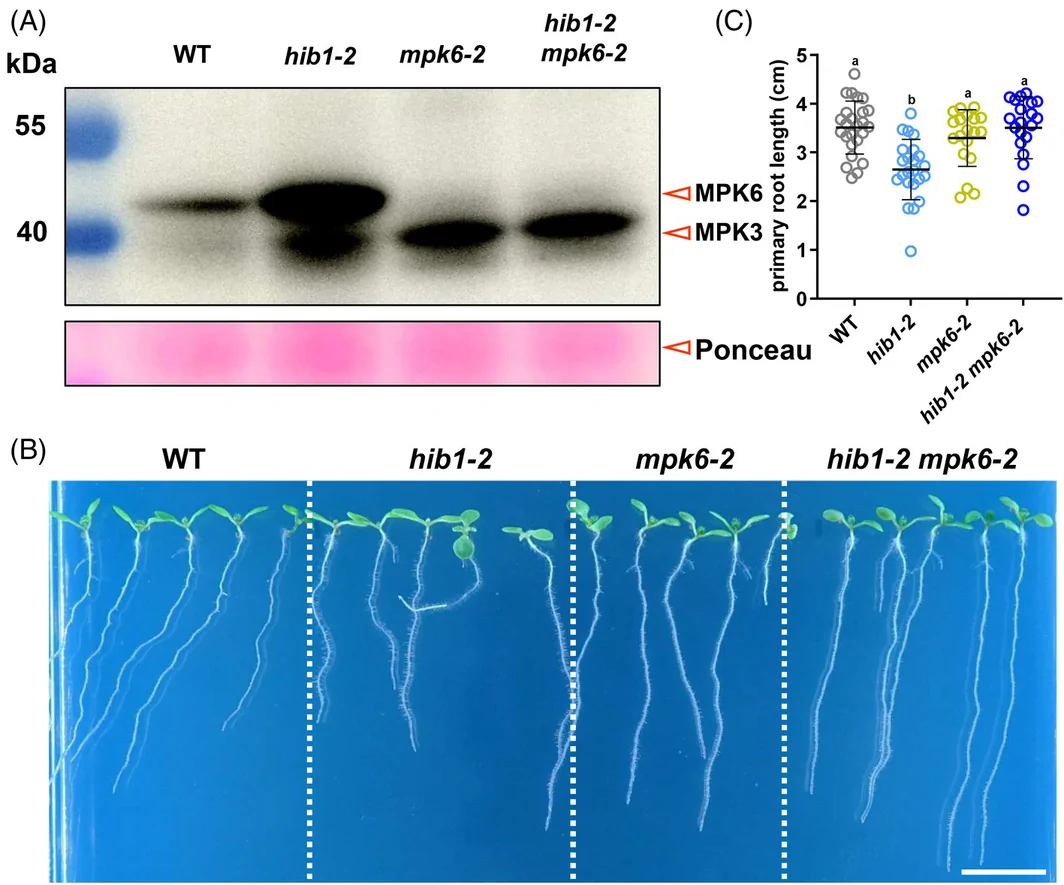
HIB1 regulates root gravitropic growth via MPK6 signaling. (A) Western blot showing increased MPK activity in the *hib1‐2* mutant. (B, C) *mpk6‐2* mutation restored the root gravitropic growth defects of the *hib1‐2* mutant. (B) Representative images showing the morphological phenotypes of the roots of 7‐day‐old WT, *hib1‐2*, *mpk6‐2*, and *hib1‐2 mpk6‐2* seedlings grown on Murashige and Skoog (MS) media. Scale bars, 1 cm. (C) Quantitative analysis of primary root length. *n* = 25, 25, 25, and 24 replicates for WT, *hib1‐2*, *mpk6‐2*, and *hib1‐2 mpk6‐2*, respectively (C). The data are presented as the means ± SDs, and the dots represent individual values. Different letters represent significant differences; *P* < 0.05; one‐way anova with Tukey's multiple comparison test.

Further split‐luciferase complementation (LCA) assays confirmed that HIB1 cannot interact directly with MPK3 and MPK6 (Figure S15D,E). Thus, how MPK6 participates in the HIB1‐mediated regulatory pathway remains to be further elucidated.

## DISCUSSION

Root gravitropism plays a key role in shaping RSA. In this work, we identified *HIB1* as an essential regulatory component for root gravitropism and RSA. By combining genetic, cell biological, and biochemical analyses, we found that HIB1 regulates the PM localization of PIN auxin transporters and the subsequent distribution of auxin in root tips via MPK6 signaling. Notably, *HIB1* is required for the redistribution of auxin during the root gravitropic response. Therefore, our work has established an HIB1‐MPK6 regulatory pathway for root gravitropism and development. Notably, increased MPK6 activity was detected in the *hib1‐2* mutant, which is in line with the genetic finding that the *mpk6‐2* mutation rescues the root growth defects of *hib1‐2*. However, how *HIB1* deficiency leads to increased MPK6 activity awaits further investigation, and an indirect regulatory mechanism might be involved.

Sequence alignment of HIB1 with closely related S‐Domain‐RLKs revealed that the V66 residue is not broadly conserved, highlighting its functional importance. A non‐conserved residue whose alteration causes severe developmental phenotypes (as seen in both the V66I point mutant and the T‐DNA null allele) likely occupies a critical structural or regulatory hotspot. Nevertheless, whether V66 constitutes a critical interface for specific ligand binding or receptor activation remains to be experimentally verified. Future work will prioritize identifying potential binding ligands of HIB1 and dissecting the function of residue Val66 within this signaling module, which will advance our understanding of the upstream environmental or endogenous signaling that shapes RSA.

We note that HIB1 is predominantly expressed in the LRC and epidermis, but its spatially restricted expression cannot fully explain its regulatory effects on the development of other tissue types. This phenomenon is often mediated via non‐cell‐autonomous signaling pathways within plant roots (Sparks et al., 2013; Van Norman et al., 2011). Two complementary mechanisms can explain how HIB1 modulates PIN1 and PIN3 in the stele. First, weak but detectable HIB1 signals can also be observed in root stele cells. Low‐level HIB1 accumulation in the stele provides a cell‐autonomous basis to locally modulate PIN1 and PIN3 in vascular tissues. Second, non‐cell‐autonomous signaling originating from the LRC and some epidermis mediated by HIB1 constitutes a major regulatory route. HIB1 activates a mobile secondary signal that moves inwards via the apoplast or plasmodesmata to regulate PIN trafficking and polar localization in inner root tissues: (1) HIB1 may stimulate the generation or modification of mobile small molecules, such as ROS, Ca^2+^, or phosphorylated sugars, which can rapidly propagate to pericycle cells via plasmodesmata and directly or indirectly modulate PIN homeostasis; (2) HIB1 signaling may activate a transcription factor in outer cells. These transcription factor target genes (such as another class of secreted peptides) move into inner tissues to function. This scenario mirrors the behavior of WOX5, which is expressed in the QC and sustains distal stem cells through non‐cell‐autonomous signaling (Stahl et al., 2009, 2013); (3) HIB1 may modulate local auxin distribution and signaling in the LRC and epidermis. As a polarly transported signaling molecule, auxin propagates local fluctuations rapidly toward the inner pericycle and vascular tissues, triggering reorganization of PIN homeostasis (Billou et al., 2005). Regardless of these hypotheses, how HIB1 performs non‐cell‐autonomous regulation still awaits further investigation.

Currently, little direct evidence of non‐cell‐autonomous regulation of auxin transport and responses in root development is known, although multiple canonical examples of non‐cell‐autonomous governing of root development have been characterized. RGF/GLV peptides perceived by RGIs in LRC are known to regulate root gravitropic growth by modulating PIN2 turnover (Whitford et al., 2012). CLE40 travels from differentiated CCs to columella stem cells to suppress *WOX5* expression (Stahl et al., 2009, 2013). Since WOX5 regulates auxin biosynthesis and transport to maintain stem cell homeostasis (Tian et al., 2014), this pathway suggests that CLE40 exerts non‐cell‐autonomous control on auxin transport. While SHORT‐ROOT (SHR), a canonical example of non‐cell‐autonomous regulation, can move from the root vascular tissue into the surrounding cell layer to regulate cell fate choices (Nakajima et al., 2001), robust evidence linking SHR activity to the regulation of auxin transport and responses remains insufficient. Collectively, our work provides robust evidence for non‐cell‐autonomous regulation of auxin transport and responses in plant roots.

Several characterized small peptides, notably the RGF/GLV family, are known to regulate root gravitropic growth by modulating PIN2 turnover (Whitford et al., 2012). GLV6/10 and RGF1 can activate MPK3/MPK6 to regulate LR formation (Fernandez et al., 2020) and maintain root stem cell homeostasis (Lu et al., 2020; Shao et al., 2020), respectively. Importantly, the receptors for these peptides, such as RGIs, are prominently expressed in the root tip (Ou et al., 2016; Whitford et al., 2012), a pattern resembling the expression domain of HIB1. In addition, CLE40 is a secreted peptide expressed in differentiated CCs (Stahl et al., 2009). After secretion, it moves short distances to reach columella stem cells and the distal root cap, where it is perceived by the receptor kinases ACR4 and CLV1 to modulate *WOX5* expression and stem cell homeostasis (Stahl et al., 2009, 2013), representing a canonical example of non‐cell‐autonomous signaling. Secreted CLE11/12/13 peptides are expressed in the root cap (Li et al., 2026). The perception of these peptides by CIK1/4 receptor kinases modulates FEZ transcription to promote the division of columella stem cells, thereby supporting proper columella root cap formation (Li et al., 2026). CLE11/12/13 peptides are also perceived by BAM1/2 receptor kinases to promote phloem initiation via non‐cell‐autonomous regulation (Zhang et al., 2024). In future work, we will focus on identifying this unknown ligand for HIB1, with CLE/RGF peptides and their homologs as candidates, which may represent a novel small peptide or mobile signal that orchestrates root development from the LRC.

## MATERIALS AND METHODS

### Plant materials and growth conditions

The *A. thaliana* (L.) line Columbia‐0 (WT), T‐DNA insertion mutant *hib1‐2* (*SALK_076637*), EMS‐mutagenesis IBU hypersensitive mutant *hib1‐1* in the *PIN2::PIN2‐GFP* background, *mpk6‐2* (*SALK_073907*) and *mpk3‐1* (*SALK_151594*) were used in this research. Additional transgenic lines expressing the *PIN* fluorescent reporters *PIN1::PIN1‐GFP* (Benková et al., 2003), *PIN2::PIN2‐GFP* (Billou et al., 2005), *PIN3::PIN3‐GFP* (Zadnikova et al., 2010), *PIN4::PIN4‐GFP* (Friml et al., 2002), *PIN7::PIN7‐GFP* (Robert et al., 2013; Zadnikova et al., 2010), and the auxin response reporter *DR5rev::GFP* (Friml et al., 2003) were crossed with *hib1‐2* to study auxin transport and distribution. For all the lines, the homozygous F_3_ progeny were selected and used for analysis. Comparisons were made among the WT, *hib1‐2*, and other transgenic combinations to evaluate the effects of the *hib1‐2* mutation on auxin‐related processes. A list of all the plant lines, including mutants, reporters and crosses, is provided in Table S1. A list of all of the reagents is provided in Table S2. A list of the primers used to genotype the T‐DNA mutants is provided in Table S3.

The plants were grown in soil or on MS media supplemented with 1% (w/v) sucrose and 0.8% (w/v) agar under controlled growth conditions (16 h light/8 h dark, 22°C) in growth chambers. The seeds were surface sterilized with 75% ethanol for 15 min, followed by stratification at 4°C for 48 h before being transferred to the growth chambers. For the stress experiments, MS medium was supplemented with IBU at various concentrations (0, 10, 20, 30, 40, and 50 μM).

### Forward genetic screening and next‐generation sequencing

EMS mutagenesis was performed, and seeds from *PIN2::PIN2‐GFP* background plants were mutagenized with 0.2% (v/v) EMS. M2 populations were screened on MS media supplemented with 40 μM IBU for determination of hypersensitive phenotypes. Candidate mutants displaying hypersensitivity (*hib1‐1*) were selected for further analysis.

For NGS mapping analysis, the genomic DNA from the *hib1‐1* mutant and WT was subjected to whole‐genome sequencing via the Illumina platform. SNP calling and mapping were performed via Bowtie2 and GATK to identify the causative mutation.

### Allelic test

Allelic tests were performed by crossing *hib1‐2* and *hib1‐1*, and the *hib1‐2 hib1‐1* F_1_ plants were analyzed. WT, *hib1‐2*, *hib1‐1*, and *hib1‐2 hib1‐1* F_1_ plants were treated with 20 μM IBU, after which the root growth conditions were analyzed.

### Image analysis and morphological analysis

To analyze primary root growth, 7‐ or 13‐day‐old seedlings grown on 1/2 MS plates with or without different concentrations of IBU were imaged with a camera (Sony A6000 camera). We subsequently quantified primary root length and root tip angles relative to the gravity vector via ImageJ software.

To analyze the number and growth angle of LRs, the seedlings were grown vertically on MS plates for 10 or 13 days under 16 h light/8 h dark and 22°C greenhouse conditions. Photographs were taken with a camera (Sony A6000 camera), and the LR angle was measured relative to the plumb line (gravity vector) 2 mm from the base of the root via ImageJ software. The LR number was directly counted manually.

### Gravitropic response test

For the gravitropic response assays, the seeds were germinated on vertical MS plates. After 5 days, the WT and *hib1‐2* seedlings were rotated by 90° to induce a gravitropic response. The plants were imaged at 0, 2, 4, 6, 12, and 24 h after reorientation. The root curvature and primary root length were measured via ImageJ software, and comparisons were made between WT and *hib1‐2* to assess differences in the response to gravity.

### Pharmacological treatment

For long‐term growth experiments, Arabidopsis seeds were sown on vertical plates with 1/2 MS medium supplemented with the indicated chemicals, including ibuprofen (Sigma, 375160‐5G, St. Louis, MO, USA), IAA (Aladdin, I101073‐250 mg, Shanghai, China), and IBA (Aladdin, I108273‐5 g). After 2 days of stratification, the plants were transferred to a growth chamber as described in the ‘Plant Material and Growth Conditions’ section for 7, 10, or 13 days. For short‐term treatment, 4 or 5‐day‐old seedlings were incubated in liquid 1/2 MS medium supplemented with the indicated chemicals for the indicated time course. Details are described below for each treatment or staining series. All the chemicals used in this study are listed in Table S2.

### Molecular cloning

The plasmid construct and generation of transgenic plants with 1.6‐kb promoter regions (upstream of the ATG start codon) and the coding DNA sequence of *HIB1* were amplified via PCR and cloned and inserted into pDONR‐P4P1r (Gateway; Invitrogen, Waltham, MA, USA) and pENTR D‐TOPO, respectively, to generate entry clones via homologous recombination. The fragments from the entry clones were subsequently cloned and inserted into the Arabidopsis expression vectors pBIB‐BASTA‐GUS‐GWR, pB7FWG2, and pH7m34GW via an LR recombination reaction. A list of all of the reagents, including restriction enzymes and Gateway clonases, is provided in Table S2. A list of the primers used for molecular cloning is provided in Table S3. A list of the resulting plasmids from this study is provided in Table S4.

The binary vector was subsequently transformed into *Agrobacterium tumefaciens* GV3101. Arabidopsis plants were subsequently transformed via the floral dip method (Clough & Bent, 1998).

### GUS staining

After the transgenic plants were obtained, 8 days *pHIB1::GUS* seedlings from at least three independent transgenic lines were analyzed. A representative line showing consistent GUS staining results was used for recording. The representative line was further analyzed for GUS staining at a later stage.

The plant materials were immersed in GUS solution buffer containing 2 mg ml^−1^ X‐Gluc, 0.5 mM K_3_Fe(CN)_6_, 0.5 mM K_4_Fe(CN)_6_, 0.1 mM sodium phosphate, 0.1 mM Na_2_EDTA, 1% (v/v) Triton X‐100, 10% (v/v) methanol, and 2.5% (v/v) DMSO. The mixture was incubated overnight at 37°C (Peng et al., 2024). After staining, the seedlings were destained with 90 (v/v) and 70% (v/v) ethanol at room temperature until the chlorophyll was completely removed. Specifically, for the imaging of roots, the decolorized materials were mounted with a transparent liquid (chloral hydrate:H_2_O:glycerol = 8:3:1).

### FM4‐64 staining assays

To analyze the subcellular localization of HIB1, one lipophilic fluorescent dye, FM4‐64 (N‐(3‐triethylammoniumpropyl)‐4‐(6‐(4‐(diethylamino) phenyl) hexatrienyl) pyridinium dibromide; Invitrogen, T13320), was used. Four‐day‐old *35S::HIB1‐GFP* plants were incubated in liquid media supplemented with 1 μM FM4‐64 for 15 min before being imaged with CLSM (Zeiss LSM980).

### PI staining

For PI (Thermo Fisher Scientific, P3566, Waltham, MA, USA) staining, WT and *hib1‐2* (aged 7 days) cells were stained with PI at a final concentration of 10 mg L^−1^ in liquid 1/2 MS for 1 min before being subjected to CLSM (Zeiss LSM800). The apparent number of root meristem cells was counted between the QC and the position where the cell length was two times greater than the minimum cell length in the cortex files.

### Quantitative RT–PCR (RT–qPCR) analysis of mutant lines

RT–qPCR was used to examine the transcripts of *HIB1* in *hib1‐2* and *35S::HIB1‐GFP*, with *ACTIN7* (*AT5G09810*) as an internal reference. In detail, total RNA was extracted from the indicated tissues via TRIzol (Invitrogen), and DNase was added to digest the genomic DNA. For reverse transcriptase‐quantitative polymerase chain reaction (RT–qPCR), 1 μg of each RNA sample was reverse transcribed (Takara, Kusatsu, Shiga, Japan). The resulting cDNA of the corresponding genes and *ACTIN7* was analyzed via SYBR Premix Ex Taq (Takara) with a Bio‐Rad CFX Connect Real‐Time System. The relative transcript levels of the genes of interest were normalized to those of *ACTIN7*, which was calculated by setting the wild‐type or a certain tissue as ‘1’, and the results are presented as the average ± standard deviation (SD) from three biological replicates. The primers used are listed in Table S1.

Additionally, the T‐DNA insertion mutant *hib1‐2* (*SALK_076637*) was verified through PCR via the primer pairs *hib1‐2*‐LP/RP and *hib1‐2*‐LP/LBb1.3. For the primers, see Table S1.

### Imaging DR5 and PIN fluorescent reporters with CLSM

To determine the polar localization of PIN2 and the fluorescence intensity of PIN, *PIN1::PIN1‐GFP*, *PIN2::PIN2‐GFP*, *PIN3::PIN3‐GFP*, *PIN7::PIN7‐GFP*, *PIN1::PIN1‐GFP;hib1‐2*, *PIN2::PIN2‐GFP;hib1‐2*, *PIN3::PIN3‐GFP;hib1‐2*, and *PIN7::PIN7‐GFP;hib1‐2* seedlings were grown on 1/2 MS plates for 4 days, after which we observed PIN protein abundance and the polar localization of PIN2 via CLSM. To analyze the signal intensity of DR5, *DR5rev::GFP* and *DR5rev::GFP;hib1‐2* seedlings were grown on 1/2 MS plates with or without IBU for 4 days, and after 4 days, we observed the DR5 signal intensity via CLSM.

To analyze the effects of IBU on PIN2 recycling, 5‐day‐old *PIN2::PIN2‐GFP;hib1‐2* and *PIN2::PIN2‐GFP* seedlings grown on normal plates were transferred to DMSO‐containing liquid 1/2 MS.

To analyze the effects of IBU on DR5 signal redistribution, 5‐day‐old *DR5rev::GFP;hib1‐2* and *DR5rev::GFP* seedlings grown on normal plates were transferred to DMSO/IBU‐containing plates and turned 90°C for 4 h. The root tips were subsequently imaged by CLSM. The *DR5* signal distribution was observed via CLSM.

### Western blotting

To detect MPK6/3 phosphorylation, 100 mg of 10‐day‐old seedling roots were frozen in liquid nitrogen, ground completely, and homogenized in plant extraction buffer (20 mM Tris–HCl (pH 7.5), 150 mM NaCl, 0.5% Tween‐20, 1 mM EDTA (ethylenediaminetetraacetic acid), and 1 mM DTT (1,4‐dithiothreitol)) containing a protease inhibitor cocktail (cOmplete, Roche, Basel, Switzerland). After the addition of an equal volume of 5 × SDS (sodium dodecyl sulfate) loading buffer, the samples were boiled for 10 min, fractionated by 10% SDS–PAGE (sodium dodecyl sulfate–polyacrylamide gel electrophoresis), and transferred to a PVDF membrane by wet blotting. The membranes were incubated with an anti‐phospho‐p44/42 (Cell Signaling Technology; 1:2500, Danvers, MA, USA) antibody and then with an anti‐rabbit IgG HRP (horseradish peroxidase)‐linked secondary antibody (Cell Signaling Technology; 1:5000). HRP activity was detected by the Supersignal Western Detection Reagents (Thermo Scientific), and the cells were imaged with an Amersham ImageQuant 800 system.

### Confocal laser scanning microscopy (CLSM)

CLSM was performed via a Zeiss LSM980 microscope with a GaAsP detector (Zeiss, Oberkochen, Germany). GFP‐tagged proteins were excited via a 488 nm laser, and emission signals were captured in the 495–545 nm range. mCherry‐tagged proteins were excited via a 561 nm laser, and emission signals were captured in the 580–650 nm range. To image FM4‐64‐stained cells, a laser line of 543 nm was used for excitation, and emission light with a wavelength of 600–700 nm was collected.

Images were analyzed via ImageJ software (National Institutes of Health, Bethesda, MD, USA) for signal intensity quantification and localization analysis. Representative images were selected from at least three independent biological replicates.

### Split‐luciferase complementation assay

The cDNA sequences of HIB1 were individually cloned and inserted into nLUC, and the cDNA sequences of MPK3 and MPK6 were individually cloned and inserted into cLUC vectors. *Agrobacterium* strains harboring the respective constructs were infiltrated into tobacco leaves as previously described. At 3 days post‐infiltration, the luciferin substrate was evenly applied to the leaf surface, and luciferase activity was detected via the Amersham™ ImageQuant™ 800 imaging system. All the vectors used by Fluc are listed in Table S4.

### Quantification and statistical analysis

All experiments were repeated at least 3 times with at least 20 plants per genotype per treatment, yielding consistent results. Primary root length, the root tip angle, and the number of LRs were measured from photographic images and analyzed via ImageJ (https://imagej.nih.gov/ij/download.html) (Schneider et al., 2012). The fluorescence intensity from the CLSM images was quantified via Fiji (https://fiji.sc/) (Schindelin et al., 2012). Data visualization and statistical analysis were conducted with GraphPad Prism 8 (GraphPad Software, San Diego, CA, USA); for bending curvature analyses of roots, polar bar graphs were generated via Origin 2021 (OriginLab Corporation, Northampton, MA, USA).

## ACCESSION NUMBERS

Published sequence data from this article can be found in the Arabidopsis Genome Initiative or enBank/EMBL databases. The accession numbers are as follows: *HIB1* (*AT4G32300*), *PIN1* (*AT1G73590*), *PIN2* (*AT5G57090*), *PIN3* (*AT1G70940*), and *PIN7* (*AT1G23080*). The sequencing data have been uploaded to NCBI under the accession number PRJNA1203087.

## AUTHOR CONTRIBUTIONS

ST conceived the project and designed the experiments. MMS, CZ, MC, YP, LS, and MK performed the experiments under the supervision of ST. The project was initiated by ST in the JF laboratory. MG prepared the genomic DNA samples for NGS analysis. All the authors contributed to the data analysis. MMS, CZ, and ST wrote the manuscript with input from the other coauthors, and all the authors revised and approved the submitted version.

## CONFLICT OF INTEREST

The authors declare that they have no competing interests.

## Supporting information


**Figure S1.** The physiological effects of naproxen and NPA on the root growth and development of *PIN2::PIN2‐GFP* and *hib1‐1*.
**Figure S2.** The *hib1‐1* mutation is recessive.
**Figure S3.** Next‐generation sequencing analysis revealed a point mutation in *HIB1* in the EMS mutant *hib1‐1*.
**Figure S4.** The physiological impact of IBU on WT and *hib1‐2* root development.
**Figure S5.** The physiological effects of naproxen and NPA on the root growth and development of WT and *hib1‐2*.
**Figure S6.** An allelic test revealed that *hib1‐2* is the correct candidate gene for the EMS mutant *hib1‐1*.
**Figure S7.** *pHIB1::HIB1‐mCherry* can rescue root growth and development defects in *hib1‐1*.
**Figure S8.** *pHIB1::HIB1‐mCherry* can rescue root growth and development defects in *hib1‐2*.
**Figure S9.** Physiological analysis and subcellular localization of *HIB1*.
**Figure S10.** Changes in the DR5rev::GFP signal in WT and *hib1‐2* roots under IBU and IAA treatment.
**Figure S11.** The physiological effects of IAA on the root growth and development of WT and *hib1‐2*.
**Figure S12.** IBA‐induced lateral root emergence was attenuated in both *hib1‐1* and *hib1‐2*.
**Figure S13.** Decreased expression abundance of PIN proteins in the hib1‐2 mutation.
**Figure S14.** Loss of *HIB1* disrupts PIN2 trafficking and bulk endocytosis.
**Figure S15.** Genetic and biochemical analyses revealing the functional relationship between *HIB1* and *MPK3/MPK6*.

**Table S1.** List of plant lines, including mutants and maker lines, used in this study.
**Table S2.** List of reagents used in this study.
**Table S3.** List of primers used in this study.
**Table S4.** List of plasmids used in this study.

## Data Availability

The data that support the findings of this study are available on request from the corresponding author. The data are not publicly available due to privacy or ethical restrictions.

## References

[tpj71206-bib-0001] Benková, E. , Michniewicz, M. , Sauer, M. , Teichmann, T. , Seifertová, D. , Jürgens, G. et al. (2003) Local, efflux‐dependent auxin gradients as a common module for plant organ formation. Cell, 115, 591–602. 10.1016/s0092-8674(03)00924-3 14651850

[tpj71206-bib-0002] Bi, G. & Zhou, J.M. (2017) MAP kinase signaling pathways: a hub of plant‐microbe interactions. Cell Host & Microbe, 21, 270–273. 10.1016/j.chom.2017.02.004 28279328

[tpj71206-bib-0003] Blilou, I. , Xu, J. , Wildwater, M. , Willemsen, V. , Paponov, I. , Frimi, J. et al. (2005) The PIN auxin efflux facilitator network controls growth and patterning in *Arabidopsis* roots. Nature, 433, 39–44. 10.1038/nature03184 15635403

[tpj71206-bib-0004] Casanova, J.E. (2007) Regulation of Arf activation: the Sec7 family of guanine nucleotide exchange factors. Traffic, 8(11), 1476–1485. 10.1111/j.1600-0854.2007.00634.x 17850229

[tpj71206-bib-0005] Chardin, P. & McCormick, F. (1999) Brefeldin A: the advantage of being uncompetitive. Cell, 97, 153–155. 10.1016/s0092-8674(00)80724-2 10219235

[tpj71206-bib-0006] Clough, S.J. & Bent, A.F. (1998) Floral dip: a simplified method for *Agrobacterium*‐mediated transformation of *Arabidopsis thaliana* . The Plant Journal, 16, 735–743. 10.1046/j.1365-313x.1998.00343.x 10069079

[tpj71206-bib-0007] Dievart, A. , Gottin, C. , Périn, C. , Ranwez, V. & Chantret, N. (2020) Origin and diversity of plant receptor‐like kinases. Annual Review of Plant Biology, 71, 131–156. 10.1146/annurev-arplant-073019-025927 32186895

[tpj71206-bib-0008] Du, Y. , Tejos, R. , Beck, M. , Himschoot, E. , Li, H. , Robatzek, S. et al. (2013) Salicylic acid interferes with clathrin‐mediated endocytic protein trafficking. Proceedings of the National Academy of Sciences, 110(19), 7946–7951. 10.1073/pnas.1220205110 PMC3651428 23613581

[tpj71206-bib-0009] Duggan, K.C. , Hermanson, D.J. , Musee, J. , Prusakiewicz, J.J. , Scheib, J.L. , Carter, B.D. et al. (2011) (R)‐Profens are substrate‐selective inhibitors of endocannabinoid oxygenation by COX‐2. Nature Chemical Biology, 7, 803–809. 10.1038/nchembio.663 PMC3298755 22053353

[tpj71206-bib-0010] Fendrych, M. , Akhmanova, M. , Merrin, J. , Glanc, M. , Hagihara, S. , Takahashi, K. et al. (2018) Rapid and reversible root growth inhibition by TIR1 auxin signalling. Nature Plants, 4(7), 453–459. 10.1038/s41477-018-0190-1 PMC6104345 29942048

[tpj71206-bib-0011] Fernandez, A.I. , Vangheluwe, N. , Xu, K. , Jourquin, J. , Claus, L.A.N. , Morales‐Herrera, S. et al. (2020) GOLVEN peptide signalling through RGI receptors and MPK6 restricts asymmetric cell division during lateral root initiation. Nature Plants, 6(5), 533–543. 10.1038/s41477-020-0645-z 32393883

[tpj71206-bib-0012] Friml, J. , Benková, E. , Blilou, I. , Wisniewska, J. , Hamann, T. , Ljung, K. et al. (2002) AtPIN4 mediates sink‐driven auxin gradients and root patterning in *Arabidopsis* . Cell, 108, 661–673. 10.1016/s0092-8674(02)00656-6 11893337

[tpj71206-bib-0013] Friml, J. , Vieten, A. , Sauer, M. , Weijers, D. , Schwarz, H. , Hamann, T. et al. (2003) Efflux‐dependent auxin gradients establish the apical‐basal axis of Arabidopsis. Nature, 426, 147–153. 10.1038/nature02085 14614497

[tpj71206-bib-0014] Furutani, M. , Hirano, Y. , Nishimura, T. , Nakamura, M. , Fukao, Y. , Hakoshima, T. et al. (2020) Polar recruitment of RLD by LAZY1‐like protein during gravity signaling in root branch angle control. Nature Communications, 11, 76. 10.1038/s41467-019-13729-7 PMC6941992 31900388

[tpj71206-bib-0015] Geldner, N. , Anders, N. , Wolters, H. , Keicher, J. , Kornberger, W. , Muller, P. et al. (2003) The *Arabidopsis* GNOM ARF‐GEF mediates endosomal recycling, auxin transport, and auxin‐dependent plant growth. Cell, 112, 219–230. 10.1016/s0092-8674(03)00003-5 12553910

[tpj71206-bib-0016] Geldner, N. , Friml, J. , Stierhof, Y.D. , Jürgens, G. & Palme, K. (2001) Auxin transport inhibitors block PIN1 cycling and vesicle trafficking. Nature, 413, 425–428. 10.1038/35096571 11574889

[tpj71206-bib-0017] Hawley, S.A. , Fullerton, M.D. , Ross, F.A. , Schertzer, J.D. , Chevtzoff, C. , Walker, K.J. et al. (2012) The ancient drug salicylate directly activates AMP‐activated protein kinase. Science, 336, 918–922. 10.1126/science.1215327 PMC3399766 22517326

[tpj71206-bib-0018] Jung, J.K. & McCouch, S.R. (2013) Getting to the roots of it: genetic and hormonal control of root architecture. Frontiers in Plant Science, 4, 186. 10.3389/fpls.2013.00186 PMC3685011 23785372

[tpj71206-bib-0019] Khan, M.A. , Gemenet, D.C. & Villordon, A. (2016) Root system architecture and abiotic stress tolerance: current knowledge in root and tuber crops [Review]. Frontiers in Plant Science, 7, 1584. 10.3389/fpls.2016.01584 PMC5088196 27847508

[tpj71206-bib-0020] Kleine‐Vehn, J. , Ding, Z. , Jones, A.R. , Tasaka, M. , Morita, M.T. & Friml, J. (2010) Gravity‐induced PIN transcytosis for polarization of auxin fluxes in gravity‐sensing root cells. Proceedings of the National Academy of Sciences, 107(51), 22344–22349. 10.1073/pnas.1013145107 PMC3009804 21135243

[tpj71206-bib-0021] Kleine‐Vehn, J. , Huang, F. , Naramoto, S. , Zhang, J. , Michniewicz, M. , Offringa, R. et al. (2009) PIN auxin efflux carrier polarity is regulated by PINOID kinase‐mediated recruitment into GNOM‐independent trafficking in *Arabidopsis* . The Plant Cell, 21, 3839–3849. 10.1105/tpc.109.071639 PMC2814515 20040538

[tpj71206-bib-0022] Krecek, P. , Skupa, P. , Libus, J. , Naramoto, S. , Tejos, R. , Friml, J. et al. (2009) The PIN‐FORMED (PIN) protein family of auxin transporters. Genome Biology, 10(12), 249. 10.1186/gb-2009-10-12-249 PMC2812941 20053306

[tpj71206-bib-0023] Li, S. , Zeng, L. , Zhao, J. , Hu, C. , Zhu, Y. , Cui, Y. et al. (2026) A CLE‐CIK signalling module controls columella root cap formation in Arabidopsis. New Phytologist, 250(4), 2341–2356. 10.1111/nph.71083 41841516

[tpj71206-bib-0024] Lichterman, B.L. (2004) Aspirin: the story of a wonder drug. BMJ, 329, 1408.

[tpj71206-bib-0025] Lu, X. , Shi, H. , Ou, Y. , Cui, Y. , Chang, J. , Peng, L. et al. (2020) RGF1‐RGI1, a peptide‐receptor complex, regulates Arabidopsis root meristem development via a MAPK signalling cascade. Molecular Plant, 13(11), 1594–1607. 10.1016/j.molp.2020.09.005 32916335

[tpj71206-bib-0026] Nakajima, K. , Sena, G. , Nawy, T. & Benfey, P.N. (2001) Intercellular movement of the putative transcription factor SHR in root patterning. Nature, 413(6853), 307–311. 10.1038/35095061 11565032

[tpj71206-bib-0027] Naramoto, S. , Kleine‐Vehn, J. , Robert, S. , Fujimoto, M. , Dainobu, T. , Paciorek, T. et al. (2010) ADP‐ribosylation factor machinery mediates endocytosis in plant cells. Proceedings of the National Academy of Sciences of the United States of America, 107(50), 21890–21895. 10.1073/pnas.1016260107 PMC3003034 21118984

[tpj71206-bib-0028] Naramoto, S. , Otegui, M.S. , Kutsuna, N. , de Rycke, R. , Dainobu, T. , Karampelias, M. et al. (2014) Insights into the localization and function of the membrane trafficking regulator GNOM ARF‐GEF at the Golgi apparatus in Arabidopsis. Plant Cell, 26, 3062–3076. 10.1105/tpc.114.125880 PMC4145132 25012191

[tpj71206-bib-0029] Ogura, T. , Goeschl, C. , Filiault, D. , Mirea, M. , Slovak, R. , Wolhrab, B. et al. (2019) Root system depth in *Arabidopsis* is shaped by EXOCYST70A3 via the dynamic modulation of auxin transport. Cell, 178, 400–412. 10.1016/j.cell.2019.06.021 31299202

[tpj71206-bib-0030] Orman‐Ligeza, B. , Parizot, B. , Gantet, P.P. , Beeckman, T. , Bennett, M.J. & Draye, X. (2013) Post‐embryonic root organogenesis in cereals: branching out from model plants. Trends in Plant Science, 18, 1360–1385. 10.1016/j.tplants.2013.04.010 23727199

[tpj71206-bib-0031] Ötvös, K. , Marconi, M. , Vega, A. , Johnson, A. , Abualia, R. , Antonielli, L. et al. (2021) Modulation of root growth by nutrient‐defined fine‐tuning of polar auxin transport. The EMBO Journal, 40, e106862. 10.15252/embj.2020106862 PMC7849315 33399250

[tpj71206-bib-0032] Ou, Y. , Lu, X. , Zi, Q. , Xun, Q. , Zhang, J. , Wu, Y. et al. (2016) RGF1 INSENSITIVE 1 to 5, a group of LRR receptor‐like kinases, are essential for the perception of root meristem growth factor 1 in *Arabidopsis thaliana* . Cell Research, 26(6), 686–698. 10.1038/cr.2016.63 PMC4897188 27229312

[tpj71206-bib-0033] Overvoorde, P. , Fukaki, H. & Beeckman, T. (2010) Auxin control of root development. Cold Spring Harbor Perspectives in Biology, 2, a001537. 10.1101/cshperspect.a001537 PMC2869515 20516130

[tpj71206-bib-0034] Peng, Y. , Ji, K. , Mao, Y. , Wang, Y. , Korbei, B. , Luschnig, C. et al. (2024) Polarly localized Bro1 domain proteins regulate PIN‐FORMED abundance and root gravitropic growth in Arabidopsis. Communications Biology, 7, 1085. 10.1038/s42003-024-06747-9 PMC11374797 39232040

[tpj71206-bib-0035] Quint, M. & Gray, W.M. (2006) Auxin signaling. Current Opinion in Plant Biology, 9, 448–453. 10.1016/j.pbi.2006.07.006 PMC2424235 16877027

[tpj71206-bib-0036] Rentel, M.C. , Lecourieux, D. , Ouaked, F. , Usher, S.L. , Petersen, L. , Okamoto, H. et al. (2004) OXI1 kinase is necessary for oxidative burst‐mediated signalling in *Arabidopsis* . Nature, 427, 858–861. 10.1038/nature02353 14985766

[tpj71206-bib-0037] Richter, S. , Kientz, M. , Brumm, S. , Nielsen, M.E. , Park, M. , Gavidia, R. et al. (2014) Delivery of endocytosed proteins to the cell–division plane requires change of pathway from recycling to secretion. eLife, 3, e02131. 10.7554/eLife.02131 PMC3979144 24714496

[tpj71206-bib-0038] Robert, H.S. , Grones, P. , Stepanova, A.N. , Robles, L.M. , Lokerse, A.S. , Alonso, J.M. et al. (2013) Local auxin sources orient the apical‐basal axis in *Arabidopsis* embryos. Current Biology, 23, 2506–2512. 10.1016/j.cub.2013.09.039 24291089

[tpj71206-bib-0039] Rowlinson, S.W. , Kiefer, J.R. , Prusakiewicz, J.J. , Pawlitz, J.L. , Kozak, K.R. , Kalgutkar, A.S. et al. (2003) A novel mechanism of cyclooxygenase‐2 inhibition involving interactions with Ser‐530 and Tyr‐385. The Journal of Biological Chemistry, 278, 45763–45769. 10.1074/jbc.M305481200 12925531

[tpj71206-bib-0040] Roychoudhry, S. & Kepinski, S. (2022) Auxin in Root Development. Cold Spring Harbor Perspectives in Biology, 14, a039933. 10.1101/cshperspect.a039933 PMC9121899 34312248

[tpj71206-bib-0041] Sabatini, S. , Beis, D. , Wolkenfelt, H. , Murfett, J. , Guilfoyle, T. , Malamy, J. et al. (1999) An auxin‐dependent distal organizer of pattern and polarity in the Arabidopsis root. Cell, 99(5), 463–472. 10.1016/s0092-8674(00)81535-4 10589675

[tpj71206-bib-0042] Salehin, M. , Bagchi, R. & Estelle, M. (2015) SCF TIR1/AFB‐based auxin perception: mechanism and role in plant growth and development. The Plant Cell, 27, 9–19. 10.1105/tpc.114.133744 PMC4330579 25604443

[tpj71206-bib-0043] Schindelin, J. , Arganda‐Carreras, I. , Frise, E. , Kaynig, V. , Longair, M. , Pietzsch, T. et al. (2012) Fiji: an open‐source platform for biological‐image analysis. Nature Methods, 9, 676–682. 10.1038/nmeth.2019 PMC3855844 22743772

[tpj71206-bib-0044] Schneider, C.A. , Rasband, W.S. & Eliceiri, K.W. (2012) NIH image to ImageJ: 25 years of image analysis. Nature Methods, 9, 671–675. 10.1038/nmeth.2089 PMC5554542 22930834

[tpj71206-bib-0045] Shao, Y. , Yu, X. , Xu, X. , Li, Y. , Yuan, W. , Xu, Y. et al. (2020) The YDA‐MKK4/MKK5‐MPK3/MPK6 Cascade functions downstream of the RGF1‐RGI ligand–receptor pair in regulating mitotic activity in root apical meristem. Molecular Plant, 13(11), 1608–1623. 10.1016/j.molp.2020.09.004 32916336

[tpj71206-bib-0046] Shiu, S.H. & Bleecker, A.B. (2003) Expansion of the receptor‐like kinase/Pelle gene family and receptor‐like proteins in *Arabidopsis* . Plant Physiology, 132, 530–543. 10.1104/pp.103.021964 PMC166995 12805585

[tpj71206-bib-0047] Sparks, E. , Wachsman, G. & Benfey, P.N. (2013) Spatiotemporal signalling in plant development. Nature Reviews Genetics, 14(9), 631–644. 10.1038/nrg3541 PMC3870141 23949543

[tpj71206-bib-0048] Stahl, Y. , Grabowski, S. , Bleckmann, A. , Kühnemuth, R. , Weidtkamp‐Peters, S. , Pinto, K.G. et al. (2013) Moderation of Arabidopsis root stemness by CLAVATA1 and ARABIDOPSIS CRINKLY4 receptor kinase complexes. Current Biology, 23(5), 362–371. 10.1016/j.cub.2013.01.045 23394827

[tpj71206-bib-0049] Stahl, Y. , Wink, R.H. , Ingram, G.C. & Simon, R. (2009) A signalling module controlling the stem cell niche in Arabidopsis root meristems. Current Biology, 19(11), 909–914. 10.1016/j.cub.2009.03.060 19398337

[tpj71206-bib-0050] Steinmann, T. , Geldner, N. , Grebe, M. , Mangold, S. , Jackson, C.L. , Paris, S. et al. (1999) Coordinated polar localization of auxin efflux carrier PIN1 by GNOM ARF GEF. Science, 286(5438), 316–318. 10.1126/science.286.5438.316 10514379

[tpj71206-bib-0051] Swarup, R. , Kramer, E.M. , Perry, P. , Knox, K. , Leyser, H.M.O. , Haseloff, J. et al. (2005) Root gravitropism requires lateral root cap and epidermal cells for transport and response to a mobile auxin signal. Nature Cell Biology, 7(11), 1057–1065. 10.1038/ncb1316 16244669

[tpj71206-bib-0052] Szepesi, Á. (2020) Halotropism: phytohormonal aspects and potential applications. Frontiers in Plant Science, 11, 571025. 10.3389/fpls.2020.571025 PMC7527526 33042187

[tpj71206-bib-0053] Tan, S. , Di Donato, M. , Glanc, M. , Zhang, X. , Klíma, P. , Liu, J. et al. (2020) Non‐steroidal anti‐inflammatory drugs target TWISTED DWARF1‐regulated actin dynamics and auxin transport‐mediated plant development. Cell Reports, 33, 108463. 10.1016/j.celrep.2020.108463 33264621

[tpj71206-bib-0054] Tan, S. , Luschnig, C. & Friml, J. (2021) Pho‐view of auxin: reversible protein phosphorylation in auxin biosynthesis, transport and signalling. Molecular Plant, 14, 151–165. 10.1016/j.molp.2020.11.004 33186755

[tpj71206-bib-0055] Tian, H. , Wabnik, K. , Niu, T. , Li, H. , Yu, Q. , Pollmann, S. et al. (2014) WOX5–IAA17 feedback circuit‐mediated cellular auxin response is crucial for the patterning of root stem cell niches in Arabidopsis. Molecular Plant, 7(2), 277–289. 10.1093/mp/sst118 23939433

[tpj71206-bib-0056] Van Norman, J.M. , Breakfield, N.W. & Benfey, P.N. (2011) Intercellular communication during plant development. The Plant Cell, 23(3), 855–864. 10.1105/tpc.111.082982 PMC3082268 21386031

[tpj71206-bib-0057] Vane, J.R. & Botting, R.M. (2003) The mechanism of action of aspirin. Thrombosis Research, 110, 255–258. 10.1016/s0049-3848(03)00379-7 14592543

[tpj71206-bib-0058] Vanneste, S. & Friml, J. (2009) Auxin: a trigger for change in plant development. Cell, 136, 1005–1016. 10.1016/j.cell.2009.03.001 19303845

[tpj71206-bib-0059] Wang, H.‐Z. , Yang, K.‐Z. , Zou, J.‐J. , Zhu, L.‐L. , Xie, Z.D. , Morita, M.T. et al. (2015) Transcriptional regulation of PIN genes by FOUR LIPS and MYB88 during Arabidopsis root gravitropism. Nature Communications, 6(1), 8822. 10.1038/ncomms9822 PMC4673497 26578169

[tpj71206-bib-0060] Wang, J.‐W. , Wang, L.‐J. , Mao, Y.‐B. , Cai, W.‐J. , Xue, H.‐W. & Chen, X.‐Y. (2005) Control of root cap formation by microRNA‐targeted auxin response factors in Arabidopsis. The Plant Cell, 17(8), 2204–2216. 10.1105/tpc.105.033076 PMC1182483 16006581

[tpj71206-bib-0061] Wang, X. , Chen, M. , Li, J. , Kong, M. & Tan, S. (2024) The SCOOP‐MIK2 immune pathway modulates Arabidopsis root growth and development by regulating PIN‐FORMED abundance and auxin transport. The Plant Journal, 120(1), 318–334. 10.1111/tpj.16988 39162107

[tpj71206-bib-0062] Whitford, R. , Fernandez, A. , Tejos, R. , Pérez, A.C. , Kleine‐Vehn, J. , Vanneste, S. et al. (2012) GOLVEN secretory peptides regulate auxin carrier turnover during plant gravitropic responses. Developmental Cell, 22(3), 678–685. 10.1016/j.devcel.2012.02.002 22421050

[tpj71206-bib-0063] Xia, J. , Kong, M. , Yang, Z. , Sun, L. , Peng, Y. , Mao, Y. et al. (2023) Chemical inhibition of Arabidopsis PIN‐FORMED auxin transporters by the anti‐inflammatory drug naproxen. Plant Communications, 6, 100632. 10.1016/j.xplc.2023.100632 PMC10721474 37254481

[tpj71206-bib-0064] Yin, M.‐J. , Yamamoto, Y. & Gaynor, R.B. (1998) The anti‐inflammatory agents aspirin and salicylate inhibit the activity of IκB kinase‐β. Nature, 396(6706), 77–80. 10.1038/23948 9817203

[tpj71206-bib-0065] Zadnikova, P. , Petrasek, J. , Marhavy, P. , Raz, V. , Vandenbussche, F. , Ding, Z. et al. (2010) Role of PIN‐mediated auxin efflux in apical hook development of *Arabidopsis thaliana* . Development, 137, 607–617. 10.1242/dev.041277 20110326

[tpj71206-bib-0066] Zhang, H. , Wang, Q. , Blanco‐Touriñán, N. & Hardtke, C.S. (2024) Antagonistic CLE peptide pathways shape root meristem tissue patterning. Nature Plants, 10(12), 1900–1908. 10.1038/s41477-024-01838-1 39468296

[tpj71206-bib-0067] Zhang, Y. , He, P. , Ma, X. , Yang, Z. , Pang, C. , Yu, J. et al. (2019) Auxin‐mediated statolith production for root gravitropism. New Phytologist, 224(2), 761–774. 10.1111/nph.15932 31111487

