# Supplementary material for: An S‐domain receptor‐like kinase regulates auxin distribution and root gravitropism via MPK signaling in Arabidopsis
Source: Plant J. 2026 Nov 5;128(3):e71206. doi: 10.1111/tpj.71206 (PMC13740653; doi:10.1111/tpj.71206)
Supplement: Supplementary file 1 [file TPJ-128-0-s001.docx]

**Supplementary Table 1. List of plant lines, including mutants and maker lines, used in this study.**

| ***Arabidopsis* lines** | **Source** | **Identifier** |
| --- | --- | --- |
| Col-0 | N/A | N/A |
| *hib1-1* | This study | N/A |
| *hib1-2* | [1] | SALK_076637 |
| *mpk6-2* | Prof. Juan Xu | SALK_073907 |
| *mpk3-1* | [2] | SALK_151594 |
| *hib1-2 mpk6-2* | This study | N/A |
| *hib1-2 mpk3-1* | This study | N/A |
| *hib1-1 hib1-2* F1 | This study | N/A |
| *PIN2::PIN2-GFP; hib1-1* F1 | This study | N/A |
| *PIN1::PIN1-GFP* | [3] | N/A |
| *PIN2::PIN2-GFP* | [4] | N/A |
| *PIN3::PIN3-GFP* | [5] | N/A |
| *PIN7::PIN7-GFP* | [6] | N/A |
| *DR5rev::GFP* | [7] | N/A |
| *PIN1::PIN1-GFP; hib1-2* | This study | N/A |
| *PIN2::PIN2-GFP; hib1-2* | This study | N/A |
| *PIN3::PIN3-GFP; hib1-2* | This study | N/A |
| *DR5rev::GFP; hib1-2* | This study | N/A |
| *p35S::HIB1-GFP* in Col-0 | This study | N/A |
| *pHIB1::GUS* in Col-0 | This study | N/A |
| *pHIB1:: HIB1 -mCherry* in *hib1-2* | This study | N/A |
| *pHIB1:: HIB1 -mCherry* in *hib1-1* | This study | N/A |

**Supplementary Table 2. List of reagents used in this study.**

| Reagent or Resource | Source | Identifier |
| --- | --- | --- |
| Murashige & Skoog Basal Medium including vitamins | Duchefa | Cat. # M0222.0050 |
| Hygromycin | Solarbio | Cat. # H8081 |
| Rifampicin | aladdin | Cat. #13292-46-1 |
| Kanamycin sulphate | Solarbio | Cat. #25389-94-0 |
| Spectinomycin | Inalco | Cat. #22189-32-8 |
| Ampicillin | Solarbio | Cat. #69-53-4 |
| Plant agar | Duchefa | Cat. #P1001.1000 |
| brefeldin A | Beyotime | Cat. #S1536 |
| FM4-64 | Thermo Fisher Scientific | Cat. #T13320 |
| Propidium Iodide | Thermo Fisher Scientific | Cat. #P3566 |
| cOmplete protease inhibitor cocktail | Roche | Cat. # 04693116001 |
| PhosSTOP™ | Roche | Cat. # 04906837001 |
| Ponceau S solution | Sigma-Aldrich_@_ | Cat. # P7170 |
| naproxen | Sigma | Cat. # N8280 |
| N-(1-Naphthyl) phthalamic acid | Sigma | Cat. # N12507 |
| 3-Indoleacrylic acid | Sigma | Cat. # I2273 |
| Indole-3- butanoic acid | aladdin_®_ | Cat. # 133-32-4 |
| (S)-(+)-Ibuprofen | Sigma-Aldrich | Cat. # 375160 |
| Bacterial | | |
| Trelief® 5α Chemically Competent Cell | Tsingke | Cat. # TSC-C01 |
| GV3101(pSoup-P19) Electroporation-Competent Cell | WEIDI | Cat. # AE1003S |
| Agrobacterium tumefaciens C58C1 | Lab stock | N/A |
| Antibodies | | |
| Phospho-p44/42 MAPK (Erk1/2) (Thr202/Tyr204) | Cell Signalling Technology | Cat. # 4370 |
| Kits | | |
| GeneJET Plasmid Miniprep Kit | Thermo Fisher Scientific | Cat. # K0503 |
| GeneJET Gel extraction kit | Thermo Fisher Scientific | Cat. # K0692 |
| *Dpn* I （FD703/ FD1704） | SibStar | Cat. # SE1703S |
| ClonExpress II One Step Cloning Kit | Vazyme | Cat. # C112-01 |
| FastPure Universal Plant Total RNA Isolation Kit | Vazyme | Cat. # RC411-01 |
| SuperSignal™ West Femto Maximum Sensitivity Substrate | Thermo Fisher | Cat. # 34095 |
| PrimeScript™ RT Master Mix (Perfect Real Time) | Takara | Cat. # RR036A |
| 2 × Q3 Probe qPCR Master Mix | Tolobio | Cat. # 22205 |
| D-Luciferin potassium | MCE | Cat. #HY-12591B |
| Restriction enzymes and Gateway Clonases | | |
| *Pac* I | NEB | Cat. # R0547 |
| *Sac* I | NEB | Cat. # R0156 |
| *Nde* I | NEB | Cat. # R0111 |
| *Xho* I | NEB | Cat. # R0146 |
| *Hind* III | NEB | Cat. # R3104 |
| T4 DNA Ligase Buffer | NEB | Cat. # M0202VVIAL |
| T4 DNA Ligase | NEB | Cat. # B0202SVIAL |
| DNA Ligation Kit Ver.2.1 | Takara | Cat. # 6022Q |
| Gateway™ LR Clonase™ II Enzyme mix | Thermo Fisher Scientific | Cat. # 11791100 |
| Gateway BP Clonase II Enzyme mix | Thermo Fisher Scientific | Cat. # 11789100 |
| Software and Algorithms | | |
| *Arabidopsis* Information Resource (TAIR) | N/A | https://www.arabidopsis.org/ |
| DNAMAN | DNAMAN | https://www.lynnon.com/ |
| image J | image J | https://imagej.nih.gov/ij/ |
| Fiji | Fiji | https://fiji.sc/ |
| ZEN | ZEN | https://www.zeiss.com/microscopy/en/products/software/zeiss-zen-lite.html |

**Supplementary Table 3. List of primers used in this study.**

| **Primers** | **Oligonucleotide (5’ to 3’)** | **Purpose** |
| --- | --- | --- |
| **For Genotyping T-DNA Insertional Mutants** | | |
| **Oligo Name** | **Sequence** | **Allele** |
| *hib1-2*_LP | ATGGCGGTTCTTTGATCAGA | *hib1-2* |
| *hib1-2*_RP | AGCCAAGTAGCCTCTTGTCC |  |
| *mpk6-2*-LP | CTCTGGCTCATCGCTTATGTC | *mpk6-2* |
| *mpk6-2*-RP | ATCTATGTTGGCGTTTGCAAC |  |
| *mpk3-1*-LP | ACAGCATTGACTCTGGCTCT | *mpk3-1* |
| *mpk3-1*-RP | GCGCTTATTGACAGAGGTAA |  |
| LBb1.3 | ATTTTGCCGATTTCGGAAC | SALK lines |
| **For RT-qPCR** | | |
| **Oligo Name** | **Sequence** | **Gene** |
| ACTIN7-F | CCGGTATTGTGCTCGATTCTG | *ACTIN7* |
| ACTIN7-R | TTCCCGTTCTGCGGTAGTGG | *ACTIN7* |
| HIB1q-F | GTTTTGATCTTTGTGGCCTTCCGG | *HIB1* |
| HIB1q-R | CACCGAGAAGTTATTCGTCGC | *HIB1* |
| **For Gateway Cloning** |  |  |
| **Oligo Name** | **Sequence** | **Plasmid** |
| pHIB1-F | GGCGCGCCTCCAAGTGCCAGCTGGCATCCGTA | pDONR-P4P1r-HIB1 |
| pHIB1-R | ATCGATCGGGTCTTCAAAGATGATGAAATTCCGG | pDONR-P4P1r-HIB1 |
| HIB1 w/o-F | GGGGACAAGTTTGTACAAAAAAGCAGGCTTCATGAGAGGCGTCTTCATCGTC | pDONR221-HIB1 |
| HIB1 w/o-R | GGGGACCACTTTGTACAAGAAAGCTGGGTTTCTCGGACCGGAGAGTCTCA | pDONR221-HIB1 |
| **For Split-luciferase complementation assay** | | |
| **Oligo Name** | **Sequence** | **Plasmid** |
| HIB1 nLUC-F | ACGGGGGACGAGCTCGGTACCATGAGAGGCGTCTTCATCGT | p35S::HIB1-nLUC |
| HIB1 nLUC-R | CGCGTACGAGATCTGGTCGACTCTCGGACCGGAGAGTCTCA | p35S::HIB1-nLUC |
| MPK6 cLUC-F | TCCCGGGGCGGTACCATGGACGGTGGTTCAGGTCAA | p35S::MPK6-cLUC |
| MPK6 cLUC-R | CGAAAGCTCTGCAGGTCGACCTATTGCTGATATTCTGGATTG | p35S::MPK6-cLUC |
| MPK3 cLUC-F | TCCCGGGGCGGTACCATGAACACCGGCGGTGGC | p35S::MPK3-cLUC |
| MPK3 cLUC-R | GCTCTGCAGGTCGACCTAACCGTATGTTGGATTG | p35S::MPK3-cLUC |

**Supplementary Table 4. List of plasmids used in this study.**

| **Plasmid Name** | **Purpose** | **Information** |
| --- | --- | --- |
| **Gateway Entry Vectors** | | |
| **Plasmid Name** \| **Types** \| **Resistance in *E.coli*** | | |
| pDONR221-HIB1 | Entry Vector | Kanamycin |
| pDONR-P4-P1r-pHIB1 | Entry Vector | Kanamycin |
| pDONRP2rP3-mCherry | Entry Vector | Kanamycin |
| pDONR221-GUS-stop | Entry Vector | Kanamycin |
| **Binary Vectors** | | |
| **Plasmid Name** | **Transgenic Lines** | **Resistance *in planta*** |
| pB7FWG2 | *p35S::HIB1-GFP* | Basta |
| pB7m34GW,0 | *pHIB1::HIB1-mCherry* | Basta |
| pB7m24GW3,0 | *pHIB1::GUS* | Basta |
| **Other Vectors** | | |
| **Plasmid Vectors** | **Resistance** | **Function** |
| p35S::HIB1-nLUC | Kanamycin | LCA assay |
| p35S::MPK3-cLUC | Kanamycin | LCA assay |
| p35S::MPK6-cLUC | Kanamycin | LCA assay |

**References for Supplementary Information**

1.	Wang, T., et al., *A receptor heteromer mediates the male perception of female attractants in plants.* Nature, 2016. **531**(7593): p. 241-244.

2.	Li, H., et al., *MPK3- and MPK6-Mediated ICE1 Phosphorylation Negatively Regulates ICE1 Stability and Freezing Tolerance in Arabidopsis.* Developmental Cell, 2017. **43**(5): p. 630-642.e4.

3.	Benková, E., et al., *Local, Efflux-Dependent Auxin Gradients as a Common Module for Plant Organ Formation.* Cell, 2003. **115**(5): p. 591-602.

4.	Xu, J. and B. Scheres, *Dissection of Arabidopsis ADP-RIBOSYLATION FACTOR 1 function in epidermal cell polarity.* The Plant Cell, 2005. **17**(2): p. 525-536.

5.	Zadnikova, P., et al., *Role of PIN-mediated auxin efflux in apical hook development of Arabidopsis thaliana.* Development, 2010. **137**(4): p. 607-617.

6.	Blilou, I., et al., *The PIN auxin efflux facilitator network controls growth and patterning in Arabidopsis roots.* Nature, 2005. **433**(7021): p. 39-44.

7.	Friml, J., et al., *Efflux-dependent auxin gradients establish the apical–basal axis of Arabidopsis.* Nature, 2003. **426**(6963): p. 147-153.
