# Supplementary material for: An S‐domain receptor‐like kinase regulates auxin distribution and root gravitropism via MPK signaling in Arabidopsis
Source: Plant J. 2026 Nov 5;128(3):e71206. doi: 10.1111/tpj.71206 (PMC13740653; doi:10.1111/tpj.71206)
Supplement: Supplementary file 2 [file TPJ-128-0-s002.zip › tpj71206-sup-0003-Figurecaptions.docx]

Supplementary Figure Legends

Supplementary Figure 1. The physiological effects of naproxen and NPA on the root growth and development of ***PIN2::PIN2-GFP*** and *hib1-1.*

(A) Representative images showing the morphological changes in the roots of 7-day-old EMS mutants *hib1-1* (in the *PIN2::PIN2-GFP* background) and *PIN2::PIN2-GFP* treated with different concentrations of naproxen and NPA. The seedlings were subsequently grown on MS media supplemented with various concentrations of naproxen and NPA. DMSO was used as the solvent control. Scale bars, 1 cm.

(B–C) Quantitative analysis of primary root length (B) and lateral root number (C) in hib1-1 and *PIN2::PIN2-GFP* plants treated with naproxen and NPA. Naproxen and NPA treatment resulted in significant inhibition of primary root elongation and lateral root formation in 7-day-old *PIN2::PIN2-GFP* and *hib1-1* seedlings, as well as impairment of root gravitropism. The number of lateral roots in 7-day-old *PIN2::PIN2-GFP* and *hib1-1* seedlings treated with naproxen and NPA was quantified. n = 23, 24, 19, 23, 24, 23 and 24 replicates for *PIN2::PIN2-GFP* and n = 23, 22, 23, 23, 23, 22 and 22 *hib1-1* (B); n = 45, 45, 45, 44, 44, and 44 replicates for *PIN2::PIN2-GFP;* and n=45, 45, 45, 44, 44, and 44 replicates for *hib1-1* (C). The data are presented as the means ± SDs, and the dots represent individual values. P values were determined via an unpaired t test with Welch’s correction for the indicated pairs of *PIN2::PIN2-GFP* and *hib1-1* at a specific concentration of naproxen and NPA.

(D) Root gravitropic response analysis of 7-day-old *PIN2::PIN2-GFP* and *hib1-1* seedlings treated with naproxen and NPA**.** The naproxen and NPA treatments affected root gravitropism. The polar bar charts show the root tip angles of individual seedlings, which were used to quantify the extent of gravitropic disruption. n=22, 20, and 22 for *PIN2::PIN2-GFP,* and n = 23, 21, and 20 for *hib1-1*, respectively. Each line represents the root tip angle of an individual seedling in the polar bar charts. Unpaired t tests were used to assess significant differences among means, and F tests were employed to evaluate differences in variance. DMSO served as the control.

**Supplementary Figure 2. The *hib1-1* mutation is recessive.**

(A) Representative images demonstrating the morphological changes in the roots of 7-day-old *PIN2::PIN2-GFP*, *hib1-1*, and F1 *PIN2::PIN2-GFP; hib1-1* plants grown on MS media supplemented with 40 µM IBU. DMSO was used as the solvent control. Scale bars, 1 cm.

(B) Quantitative analysis of the primary root length of *PIN2::PIN2-GFP*, *hib1-1*, and F_1_ *PIN2::PIN2-GFP; hib1-1* plants. F_1_ plants presented a wild-type phenotype and wild-type sensitivity to IBU. n = 7 and 11 replicates for *PIN2::PIN2-GFP*, n = 7 and 9 replicates for *hib1-1* and n = 8 and 11 for F_1_ plants. The data are presented as the means ± SDs, and the dots represent individual values. Different letters represent significant differences; P < 0.05; one-way ANOVA with Tukey’s multiple comparison test.

(C) Root gravitropic response analysis of 7-day-old *PIN2::PIN2-GFP*, *hib1-1*, and F_1_ *PIN2::PIN2-GFP; hib1-1* plants treated with IBU. F_1_ plants presented wild-type root gravitropism. The polar bar charts show the root tip angles of individual seedlings, which were used to quantify the extent of gravitropic disruption. n = 7 and 11 replicates for *PIN2::PIN2-GFP*, n = 7 and 9 replicates for *hib1-1* and n = 8 and 11 for F_1_ plants. Each line represents the root tip angle of an individual seedling in the polar bar charts. Unpaired t tests were used to assess significant differences among means, and F tests were employed to evaluate differences in variance. DMSO served as the control.

Supplementary Figure 3. Next-generation sequencing analysis revealed a point mutation in *HIB1* in the EMS mutant *hib1-1*.

(A) Next-generation sequencing (NGS) revealed a point mutation at position 66 in *HIB1*, where valine (Val) is substituted with isoleucine (Ile), resulting in the EMS-hypersensitive mutant. Mapping of the *HIB1* gene is illustrated.

(B) Schematic representation of the *HIB1* gene structure. The black arrowheads indicate the positions of the *HIB1* T-DNA insertions. The white boxes represent untranslated regions (UTRs), the blue boxes represent exons, and the black lines represent introns. Scale bar, 200 bp.

(C) Identification and characterization of the *hib1-2* mutant. The position of the T-DNA insertion in the *hib1-2* mutant was identified through PCR analysis via LP (left primer), RP (right primer), and LBb1.3 (T-DNA left border primer) primers. WT served as the control. The left lanes of the upper and lower panel are the DNA ladder, cropped from the same gel.

(D) RT–qPCR was applied to determine *HIB1* expression in wild-type (WT) and *hib1-2* mutant plants. The mRNA level of *ACTIN7* was set to 1. Total RNA was extracted from 7-day-old WT and *hib1-2* seedlings. The data are presented as the means ± SDs, and the dots represent individual values. P values were calculated by comparing two different plant lines via an unpaired t test with Welch’s correction.

Supplementary Figure 4. The physiological impact of IBU on WT and *hib1-2* root development.

(A) Representative images demonstrating the morphological changes in the roots of 7-day-old WT and *hib1-2* plants grown on MS media supplemented with various concentrations of IBU. DMSO was used as the solvent control. Scale bars, 1 cm.

(B–C) Quantitative analysis of primary root length (B) and lateral root number (C) in 7-day-old WT and *hib1-2* plants treated with IBU. IBU treatment significantly inhibited primary root elongation and lateral root emergence in 7-day-old WT and *hib1-2* plants and interfered with root gravitropism. The quantification of the number of lateral roots in 7-day-old WT and *hib1-2* seedlings treated with IBU is presented. n = 25, 22, 24, 22, 21 and 23 replicates for WT and n = 21, 20, 22, 22, 23 and 23 replicates for *hib1-2*, respectively (B); n=22, 22, 23, 24, 22 and 23 replicates for WT; and n =20, 22, 22, 21, 23 and 24 replicates for *hib1-2*, respectively (C). The data are presented as the means ± SDs, and the dots represent individual values. P values were determined by comparing the WT and *hib1-2* lines via an unpaired t test with Welch’s correction at a specific concentration of IBU.

(D) IBU disrupted root gravitropism in WT and *hib1-2* plants. Seven-day-old WT and *hib1-2* seedlings were grown on MS media supplemented with DMSO or various concentrations of IBU. Polar bar charts represent the root tip angles of individual plants, indicating the extent of gravitropic disruption resulting from IBU treatment. n=25, 23, and 22 for WT and n = 20, 22, and 23 for *hib1-2*, respectively. Unpaired t tests were used to assess significant differences among means, and F tests were employed to evaluate differences in variance. DMSO served as the control.

Supplementary Figure 5. The physiological effects of naproxen and NPA on the root growth and development of **WT** and *hib1-2.*

(A) Representative images showing the morphological changes in the roots of 7-day-old *hib1-2* and WT plants treated with different concentrations of naproxen and NPA. The seedlings were subsequently grown on MS media supplemented with various concentrations of naproxen and NPA. DMSO was used as the solvent control. Scale bars, 1 cm.

(B–C) Quantitative analysis of primary root length (B) and lateral root number (C) in *hib1-2* and WT plants treated with naproxen and NPA. Naproxen and NPA treatment resulted in significant inhibition of primary root elongation and lateral root formation in 7-day-old WT and *hib1-2* seedlings, as well as impairment of root gravitropism. The number of lateral roots in 10-day-old WT and *hib1-2* seedlings treated with naproxen and NPA was quantified. n = 23, 19, 21, 24, 23, 23 and 24 replicates for WT and n = 24, 23, 24, 25, 23, 24 and 24 for *hib1-2* (B); n = 45, 45, 45, 44, 44, and 44 replicates for *PIN2::PIN2-GFP;* and n=45, 45, 45, 44, 44, and 44 replicates for *hib1-1* (C). The data are presented as the means ± SDs, and the dots represent individual values. P values were determined via an unpaired t test with Welch’s correction for the indicated pairs of WT and *hib1-2* at a specific concentration of naproxen and NPA.

(D) Root gravitropic response analysis of 7-day-old WT and *hib1-2* seedlings treated with naproxen and NPA**.** The naproxen and NPA treatments affected root gravitropism. The polar bar charts show the root tip angles of individual seedlings, which were used to quantify the extent of gravitropic disruption. n=23, 19, and 23 for WT*,* and n = 24, 23, and 24 for *hib1-2*, respectively. Each line represents the root tip angle of an individual seedling in the polar bar charts. Unpaired t tests were used to assess significant differences among means, and F tests were employed to evaluate differences in variance. DMSO served as the control.

**Supplementary Figure 6. An allelic test revealed that *hib1-2* is the correct candidate gene for the EMS mutant *hib1-1*.**

(A) Representative images showing that *hib1-2 hib1-1* (F_1_) seedlings obtained by crossing *hib1-2* and hib1-1 exhibited similar morphological changes to those of *hib1-2* and *hib1-1* when treated with 20 µM IBU or grown on normal 1/2 MS media. Seven-day-old WT, *hib1-2*, *hib1-1* and h*ib1-2 hib1-1* (F_1_) plants were grown on 1/2 MS media supplemented with DMSO and IBU. DMSO served as the control. Scale bars, 1 cm.

(B) Quantitative analysis of primary root length in 7-day-old WT, *hib1-2*, *hib1-1* and *hib1-2 hib1-1* (F_1_) plants. Compared with the WT, the new F_1_ allele significantly responded to IBU, which is consistent with the findings of *hib1-2* and *hib1-1*. n = 20, 20 replicates for WT; n = 16, 16 replicates for *hib1-2*; n = 22, 18 replicates for *hib1-1*; n = 18, 18 replicates for *hib1-2 hib1-1* (F_1_). The data are presented as the means ± SDs, and the dots represent individual values. Different letters represent significant differences; P < 0.05; one-way ANOVA with Tukey’s multiple comparison test.

(C) Root gravitropism analysis of 7-day-old WT, *hib1-2*, *hib1-1* and F_1_ generation *hib1-2 hib1-1* seedlings treated with IBU. The polar bar charts show the root tip angles of individual seedlings, which were used to quantify the extent of gravitropic disruption. n= 20, 16, 22, and 18, respectively. Each line represents the root tip angle of an individual seedling in the polar bar charts. Unpaired t tests were used to assess significant differences among means, and F tests were employed to evaluate differences in variance. DMSO served as the control.

**Supplementary Figure 7. *pHIB1::HIB1-mCherry* can rescue root growth and development defects in *hib1-1*.**

(A) Representative images showing the morphological changes in the roots of 7-day-old *PIN2::PIN2-GFP*, *hib1-1*, and *pHIB1::HIB1-mCherry;hib1-1* seedlings treated with DMSO or 40 μM IBU. DMSO served as the control. Scale bar, 1 cm.

(B) Quantitative analysis of primary root length in 7-day-old WT, *hib1-1* and *pHIB1::HIB1-mCherry;hib1-1 plants*. n = 19, 30 for WT; n = 18, 30 for *hib1-1;* and n = 20, 27 for *pHIB1::HIB1-mCherry;hib1-1*. The data are presented as the means ± SDs, and the dots represent individual values. Different letters represent significant differences; P < 0.05; one-way ANOVA with Tukey’s multiple comparison test.

(C) Confocal images showing the protein localization of *pHIB1::HIB1-mCherry* in *hib1-1* seedlings. Five-day-old *pHIB1::HIB1-mCherry* plants were grown on MS media, and the roots were imaged via CLSM in the mCherry and GFP channels. Scale bar, 50 μm.

**Supplementary Figure 8. *pHIB1::HIB1-mCherry* can rescue root growth and development defects in *hib1-2*.**

(A) Representative images showing the morphological changes in the roots of 7-day-old WT, *hib1-2*, and *pHIB1::HIB1-mCherry;hib1-2* seedlings. Scale bar, 1 cm.

(B) Quantitative analysis of primary root length in 7-day-old WT, *hib1-2*, and *pHIB1::HIB1-mCherry;hib1-2* seedlings. n = 16 for WT, n = 13 for *hib1-2* and n = 17 for *pHIB1::HIB1-mCherry;hib1-2*. The data are presented as the means ± SDs, and the dots represent individual values. Different letters represent significant differences; P < 0.05; one-way ANOVA with Tukey’s multiple comparison test.

(C) Representative micrographs of the PI-stained primary root tips of 5-day-old WT and *hib1-2* seedlings. The white lines indicate the approximate position where cells begin to elongate noticeably. Scale bar, 50 μm.

(D) The number of meristematic cortex cells of 5-day-old WT and *hib1-2* plants. The data are presented as the means ± SDs, and the dots represent individual values. Unpaired t tests were used to assess significant differences among means, and F tests were employed to evaluate differences in variance. P values were calculated by comparing two different plant lines via an unpaired t test with Welch’s correction.

(E) Confocal images showing the protein localization of *pHIB1::HIB1-mCherry* in *hib1-2* seedlings. Five-day-old *pHIB1::HIB1-mCherry;hib1-2* plants were grown on MS media, and the roots were imaged via CLSM in the mCherry channel. Scale bar, 50 μm.

Supplementary Figures 9. Physiological analysis and subcellular localization of *HIB1.*

(A) Representative images of 7-day-old WT, *35S::HIB1-GFP #9-9* and *35S::HIB1-GFP #2-1* seedlings treated with DMSO, 20 μM IBU or 40 μM IBU. DMSO served as the control. Scale bar, 1 cm.

(B) Quantitative analysis of primary root length in 7-day-old WT and *35S::HIB1-GFP* seedlings. n = 20, 19, and 18 for WT; n = 16, 20, and 15 for *35S::HIB1-GFP #9-9;* and n = 16, 18, and 15 for *35S::HIB1-GFP #2-1*, respectively. The data are presented as the means ± SDs, and the dots represent individual values. Different letters represent significant differences; P < 0.05; one-way ANOVA with Tukey’s multiple comparison test.

(C) Representative images showing the subcellular localization of HIB1. HIB1-GFP was expressed in *Arabidopsis* driven by the CaMV 35S promoter. FM4-64 staining was used as a marker for PM. Scale bar, 50 µm.

(D) Colocalization analysis of fluorescence intensities for *35S::HIB1-GFP* and FM4-64 staining signals at the positions indicated by the white lines in (C).

(E) RT–qPCR was applied to determine *HIB1* expression in *35S::HIB1-GFP #9-9* and *35S::HIB1-GFP #2-1* seedlings. The mRNA level of *ACTIN7* was set to 1. Total RNA was extracted from 7-day-old WT, *35S::HIB1-GFP #9-9* and *35S::HIB1-GFP #2-1* seedlings. The data are presented as the means ± SDs, and the dots represent individual values. Different letters represent significant differences; P < 0.05; one-way ANOVA with Tukey’s multiple comparison test.

Supplementary Figure 10. Changes in the DR5rev::GFP signal in WT and *hib1-2* roots under IBU and IAA treatment.

(A) Confocal images showing DR5rev::GFP fluorescence in the root tips of WT and *hib1-2* seedlings treated with different concentrations of IBU. Five-day-old seedlings were grown continuously on DMSO/IBU-containing plates and imaged via CLSM in the GFP channel. Scale bars, 20 μm. The dotted white circles indicate the regions quantified.

(B) Quantitative analysis of GFP fluorescence intensity in *DR5rev::GFP* and *DR5rev::GFP;hib1-2* root tips with or without IBU treatment. n = 14 and 14 replicates for *DR5rev::GFP,* and n = 14 and 14 replicates for *DR5rev::GFP;hib1-2*. The data are presented as the means ± SDs, and the dots represent individual values. Different letters represent significant differences; P < 0.05; one-way ANOVA with Tukey’s multiple comparison test.

(C) Confocal images showing DR5rev::GFP fluorescence in the root tips of WT and *hib1-2* seedlings treated with different concentrations of IBU. Five-day-old seedlings were grown continuously on DMSO/IAA-containing plates and imaged via CLSM in the GFP channel. Scale bars, 20 μm. The dotted white circles indicate the regions quantified.

(D) Quantitative analysis of GFP fluorescence intensity in *DR5rev::GFP* and *DR5rev::GFP;hib1-2* root tips with or without IAA treatment. n = 10 and 11 replicates for DR5rev::GFP, and n = 11 and 7 replicates for *DR5rev::GFP;hib1-2*. The data are presented as the means ± SDs, and the dots represent individual values. Different letters represent significant differences; P < 0.05; one-way ANOVA with Tukey’s multiple comparison test.

Supplementary Figure 11. The physiological effects of IAA on the root growth and development of **WT** and *hib1-2.*

(A) Representative images showing the morphological changes in the roots of 7-day-old WT and *hib1-2* plants treated with different concentrations of IAA. The seedlings were subsequently grown on MS media supplemented with various concentrations of IAA. DMSO was used as the solvent control. Scale bars, 1 cm.

(B) Quantitative analysis of primary root length in WT and *hib1-2* plants treated with IAA. IAA treatment resulted in significant inhibition of primary root elongation and lateral root formation in 7-day-old WT and *hib1-2* seedlings, as well as impairment of root gravitropism. n = 25, 20 and 24 replicates for WT and n = 23, 24 and 21 replicates for *hib1-2*. The data are presented as the means ± SDs, and the dots represent individual values. P values were determined via an unpaired t test with Welch’s correction for the indicated pairs of WT and *hib1-2* at a specific concentration of IAA. Different letters represent significant differences; P < 0.05; one-way ANOVA with Tukey’s multiple comparison test.

(C) Root gravitropic response analysis of 7-day-old WT and *hib1-2* seedlings treated with different concentrations of IAA**.** IAA treatment cannot rescue root agravitropism in *hib1-2*. The polar bar charts show the root tip angles of individual seedlings, which were used to quantify the extent of gravitropic disruption. n = 25, 20 and 24 replicates for WT and n = 23, 24 and 21 replicates for *hib1-2*. Each line represents the root tip angle of an individual seedling in the polar bar charts. Unpaired t tests were used to assess significant differences among means, and F tests were employed to evaluate differences in variance. DMSO served as the control.

Supplementary Figure 12. **IBA-induced lateral root emergence was attenuated in both *hib1-1* and *hib1-2****.*

(A) Representative images showing the morphological changes in the number of lateral roots in 10-day-old WT and *hib1-2* plants treated with 5 µM IBA. The seedlings were subsequently grown on MS media supplemented with 5 µM IBA. DMSO was used as the solvent control. Scale bars, 1 cm.

(B) Quantitative analysis of the number of lateral roots in 10-day-old WT and *hib1-2* plants treated with 5 µM IBA. IBA-induced lateral root emergence was attenuated in *hib1-2* seedlings. n = 23 and 22 replicates for WT and n = 23 and 24 replicates for *hib1-2*. The data are presented as the means ± SDs, and the dots represent individual values. Different letters represent significant differences; P < 0.05; one-way ANOVA with Tukey’s multiple comparison test.

(C) Representative images showing the morphological changes in the number of lateral roots in 10-day-old *PIN2::PIN2-GFP* and *hib1-1* plants treated with 5 µM IBA. The seedlings were subsequently grown on MS media supplemented with 5 µM IBA. DMSO was used as the solvent control. Scale bars, 1 cm.

(D) Quantitative analysis of the number of lateral roots in 10-day-old *PIN2::PIN2-GFP* and *hib1-1* plants treated with 5 µM IBA. IBA-induced lateral root emergence was attenuated in *hib1-1* seedlings. n = 18 and 24 replicates for *PIN2::PIN2-GFP,* and n = 23 and 21 replicates for *hib1-2*. The data are presented as the means ± SDs, and the dots represent individual values. Different letters represent significant differences; P < 0.05; one-way ANOVA with Tukey’s multiple comparison test.

Supplementary Figure 13. Decreased expression abundance of PIN proteins in the hib1-2 mutation.

(A) Confocal images showing the localization of PIN1-GFP in WT and *hib1-2* seedlings. Five-day-old seedlings were grown continuously on MS media and imaged via CLSM in the GFP channel. Scale bars, 20 μm.

(C) Confocal images showing the localization of PIN3-GFP in WT and *hib1-2* seedlings. Five-day-old seedlings were grown continuously on MS media and imaged via CLSM in the GFP channel. Scale bars, 20 μm.

(E) Confocal images showing the localization of PIN7-GFP in WT and *hib1-2* seedlings. Five-day-old seedlings were grown continuously on MS media and imaged via CLSM in the GFP channel. Scale bars, 20 μm.

(B, D, F) Quantitative analysis of the GFP signal intensity in stele and columella cells. (B) n = 10 for *PIN1::PIN1-GFP* and n = 13 for *PIN1::PIN1-GFP; hib1-2*. (D) n = 10 for *PIN3::PIN3-GFP* and n = 10 for *PIN3::PIN3-GFP; hib1-2*. (F) n = 7 for *PIN7::PIN7-GFP* and n = 9 for *PIN7::PIN7-GFP; hib1-2*. The data are presented as the means ± SDs, and the dots represent individual values. p values were calculated by comparing two different plant lines via an unpaired t test with Welch’s correction.

**Supplementary Figure 14. Loss of *HIB1* disrupts PIN2 trafficking and bulk endocytosis.**

(A) Confocal images showing the localization of PIN2::PIN2-GFP in WT and *hib1-2* cells treated with DMSO or 50 μM BFA or that were cotreated with 50 μM BFA and 40 μM IBU. Five-day-old *PIN2::PIN2-GFP* and *PIN2::PIN2-GFP; hib1-2* seedlings were grown continuously on MS media and then treated with DMSO or 50 μM BFA for 30 min, after which 40 μM IBU was added to the same system for further staining for 15 min. The roots were imaged via CLSM in the GFP channel. Scale bars, 10 μm.

**(B)** The ratio of PIN2-GFP fluorescence intensity of the intracellular:PM signal was measured in WT and *hib1-2*. n = 70 for *PIN2::PIN2-GFP*, DMSO; n = 68 for *PIN2::PIN2-GFP*, +BFA; n = 39 for PIN2::PIN2-GFP, +BFA and +IBU; n = 83 for *PIN2::PIN2-GFP; hib1-2*, DMSO; n = 42 for *PIN2::PIN2-GFP; hib1-2*, +BFA; and n = 65 for *PIN2::PIN2-GFP; hib1-2*, +BFA and +IBU. The data are presented as the means ± SDs, and the dots represent individual values. Different letters represent significant differences; P < 0.05; one-way ANOVA with Tukey’s multiple comparison test.

(C) Quantitative analysis of PIN2‑GFP fluorescence intensity in root epidermal cells in *PIN2::PIN2-GFP* and *PIN2::PIN2-GFP; hib1-2* without BFA treatment is shown in Figure 6D. n = 11 for *PIN2::PIN2-GFP* and n = 12 for *PIN2::PIN2-GFP; hib1-2*. The data are presented as the means ± SDs, and the dots represent individual values. p values were calculated by comparing two different plant lines via an unpaired t test with Welch’s correction.

(D and E) Loss of *HIB1* inhibited the uptake of FM4-64. Five-day-old WT and *hib1-2* seedlings were treated with 2 μM FM4-64 for 15 min before being subjected to CLSM. (D) Confocal images showing impaired uptake of FM4-64 by *hib1-2* cells. Scale bars, 10 μm. (E) Quantification of the fluorescence intensity ratio of the intracellular:PM signal. n = 60 WT and *hib1-2* cells. The data are presented as the means ± SDs, and the dots represent individual values. p values were calculated by comparing two different plant lines via an unpaired t test with Welch’s correction.

**Supplementary Figure 15. Genetic and biochemical analyses revealing the functional relationship between *HIB1* and *MPK3/MPK6*.**
**(A) Western blot showing increased MPK activity in the *hib1-1* and *hib1-2* mutants.**

**(B and C) *mpk3-1* mutation did not restore the root gravitropic growth defects of the *hib1-2* mutant. (B) Representative images showing the morphological phenotypes of the roots of 7-day-old wild-type WT, *hib1-2* and *hib1-2 mpk3-1 #9-3, #29-4* seedlings grown on MS media. Scale bars, 1 cm. (C) Quantitative analysis of primary root length. n=19, 20, 20, and 19 replicates for WT, *hib1-2*, mpk6-2, and *hib1-2 mpk3-1 #9-3, #29-4*, respectively (C). The data are presented as the means ± SDs, and the dots represent individual values. Different letters indicate significant differences according to an unpaired t test with Welch’s correction. The data are presented as the means ± SDs, and the dots represent individual values.**

**(D and E) Split-luciferase complementation imaging (LCI) assays failed to detect direct physical interactions between HIB1 and MPK3 (D) or between HIB1 and MPK6 (E) in *Nicotiana benthamiana* epidermal cells.**
