# Supplementary material for: An S‐domain receptor‐like kinase regulates auxin distribution and root gravitropism via MPK signaling in Arabidopsis
Source: Plant J. 2026 Nov 5;128(3):e71206. doi: 10.1111/tpj.71206 (PMC13740653; doi:10.1111/tpj.71206)

Supplemental Figure 1

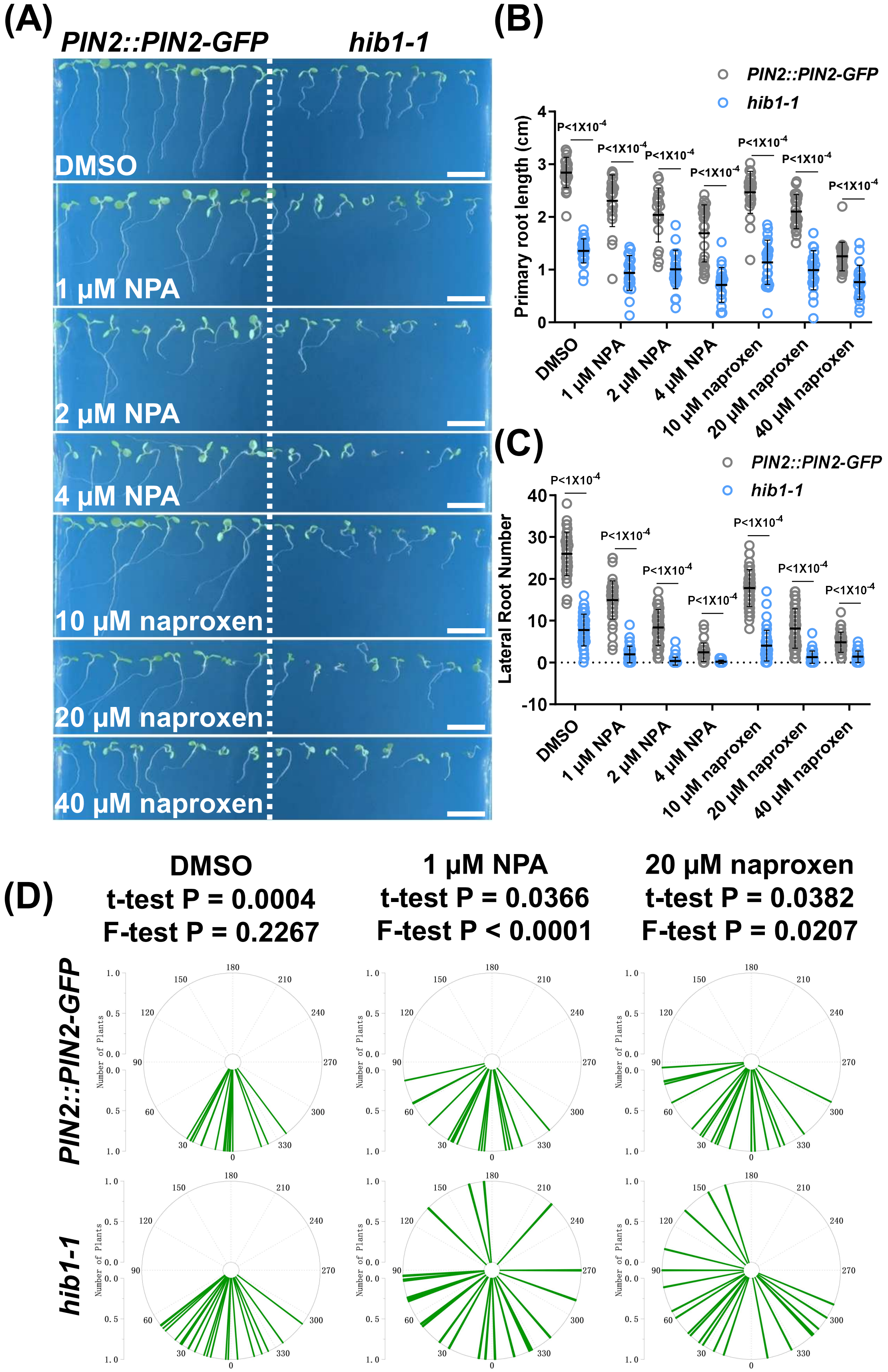

Supplemental Figure 2

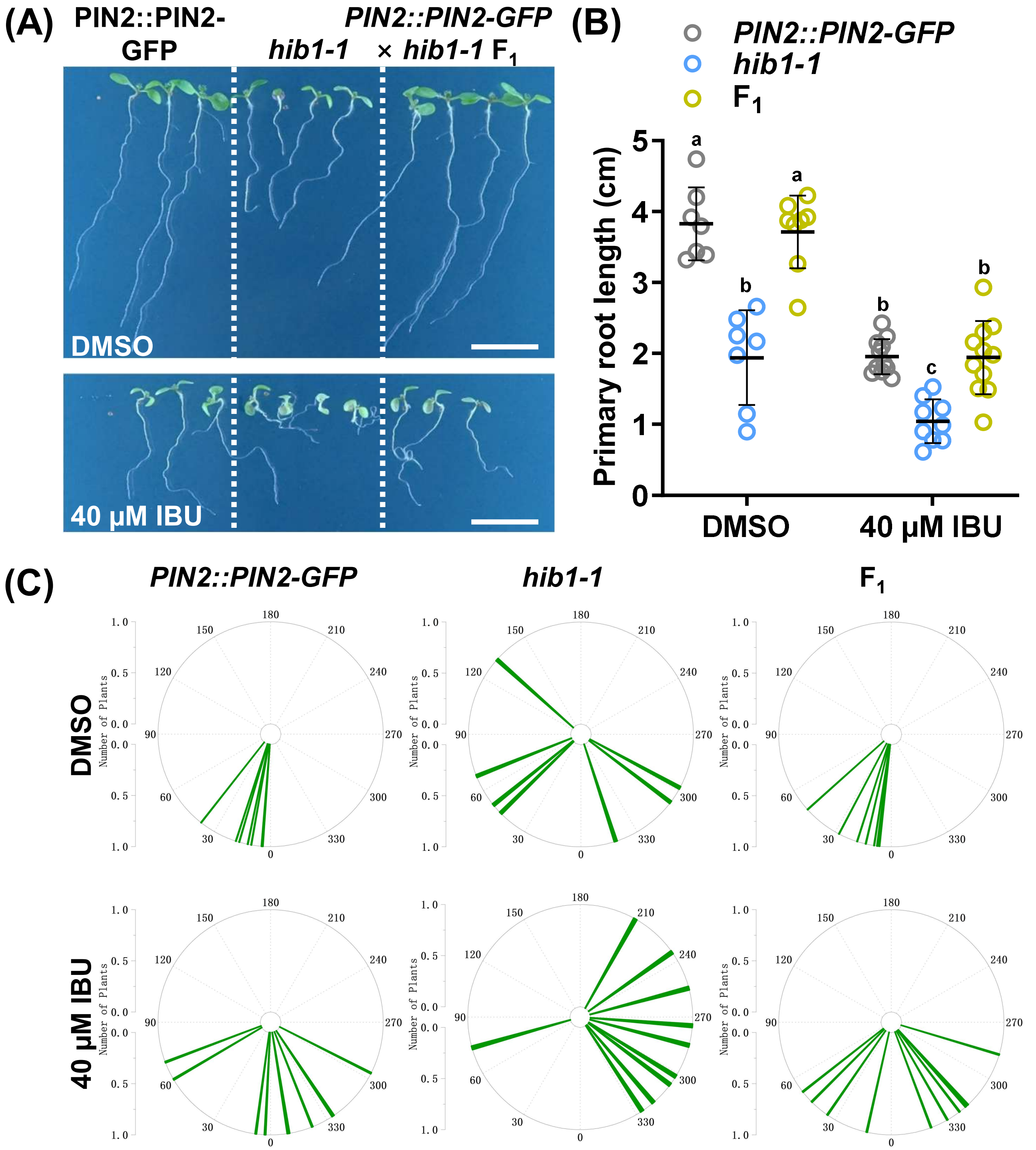

Supplemental Figure 3

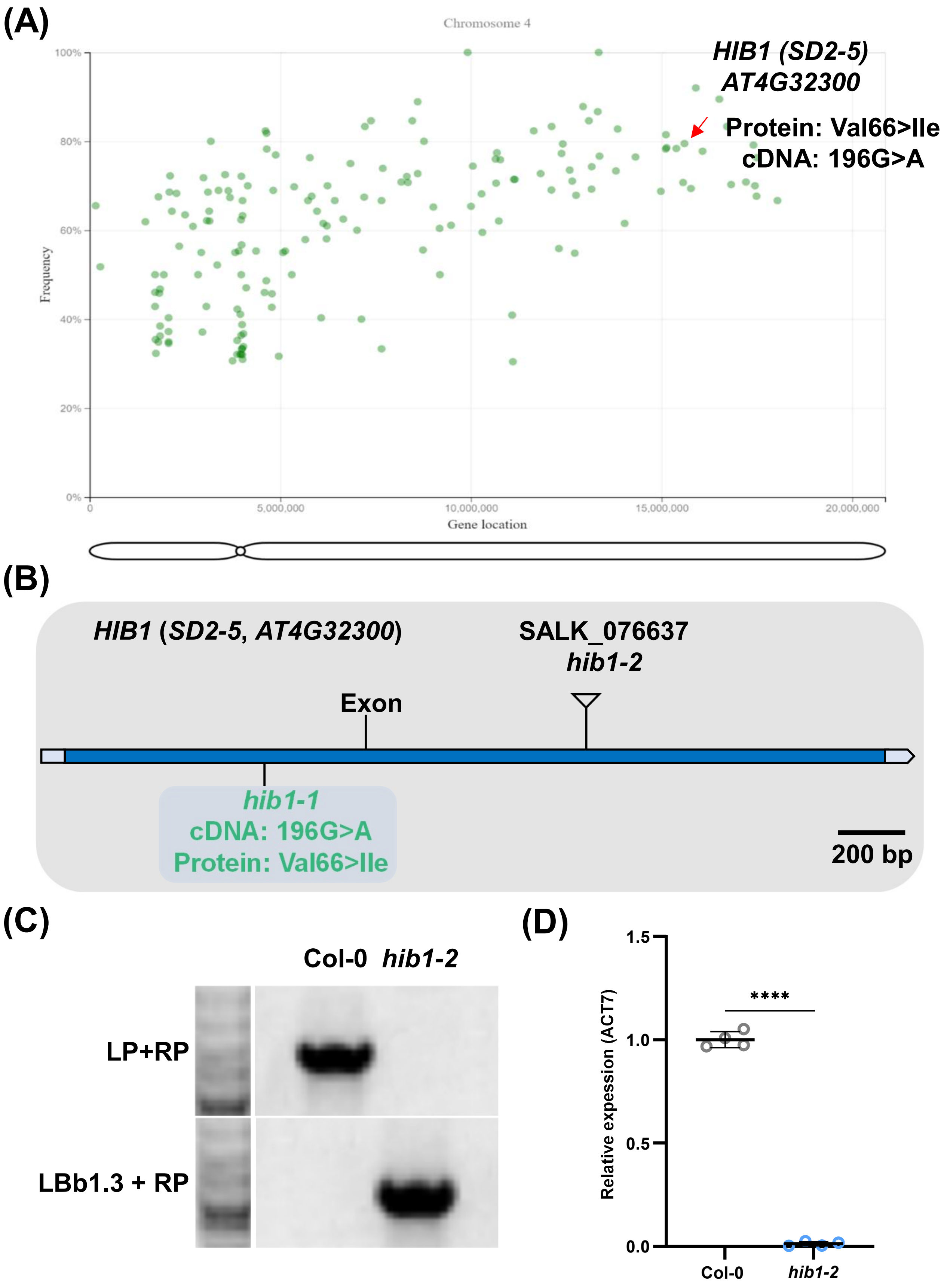

Supplemental Figure 4

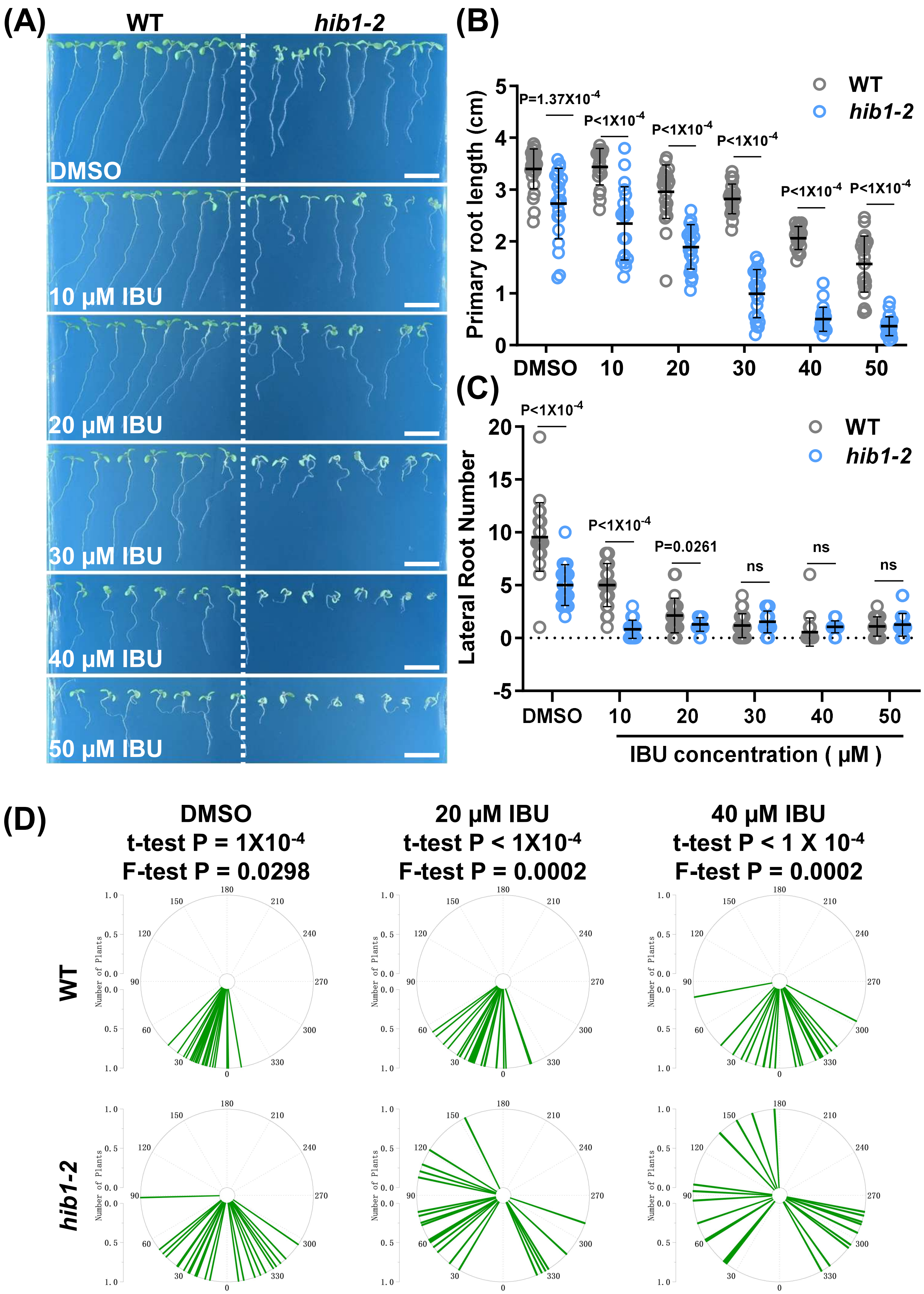

Supplemental Figure 5

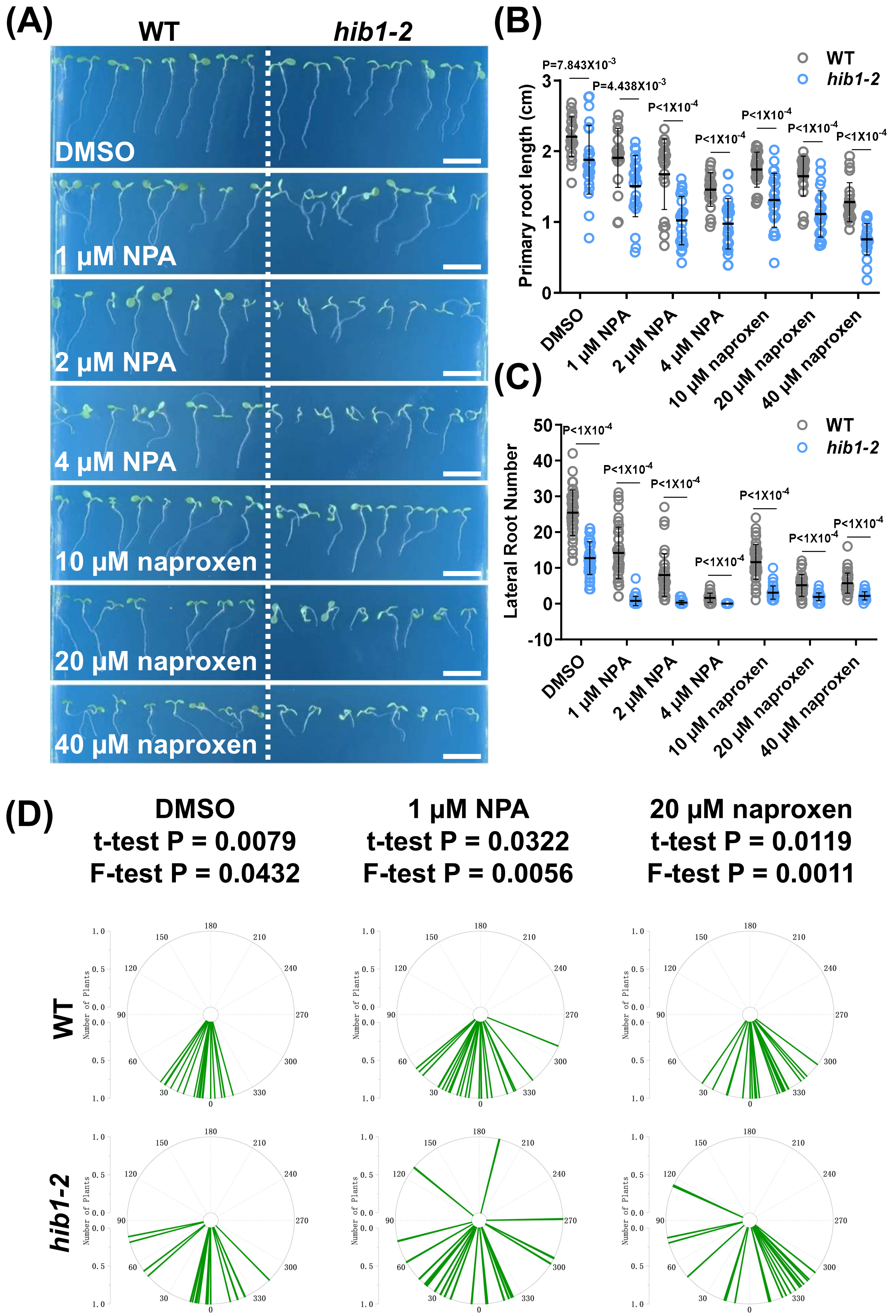

Supplemental Figure 6

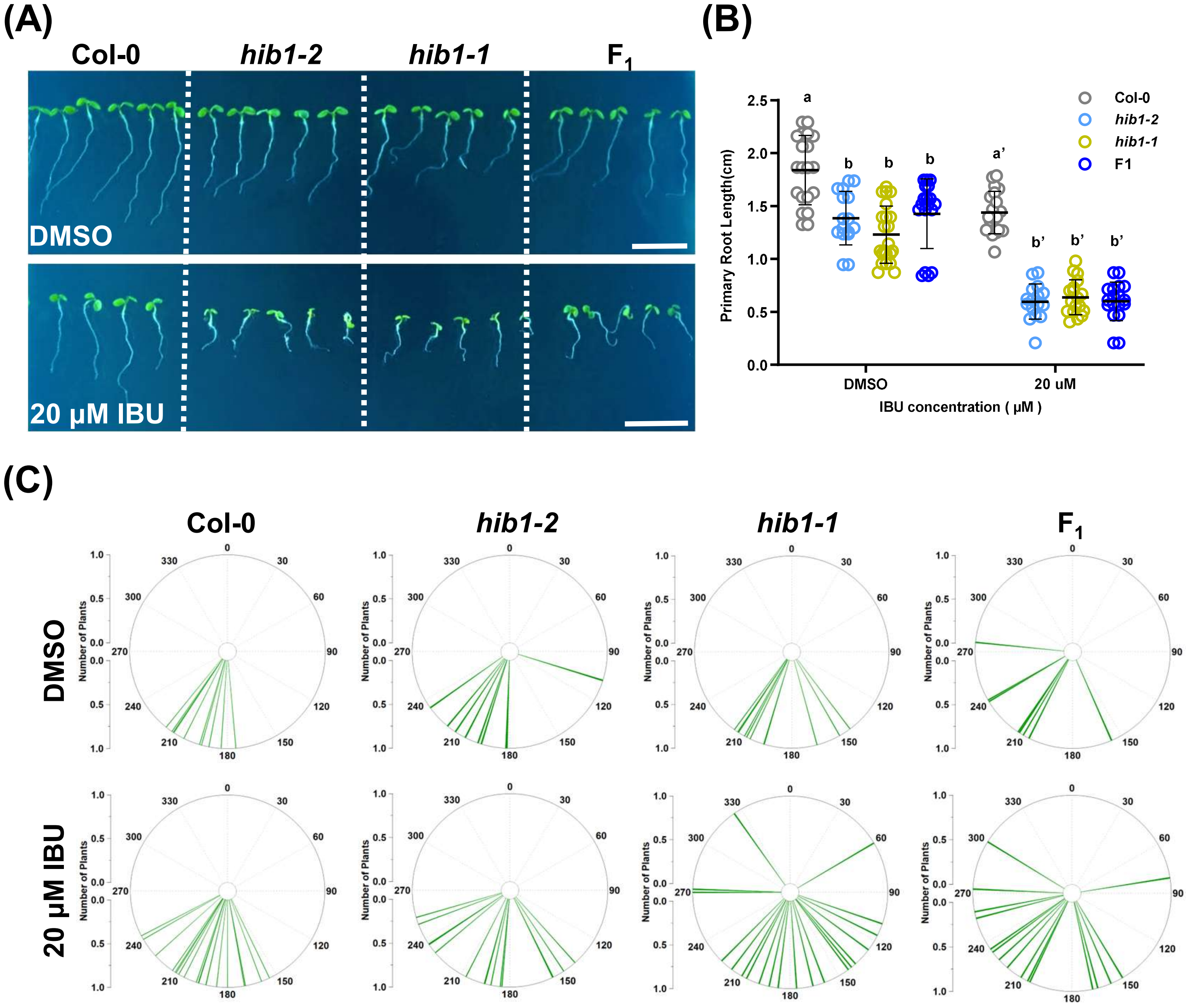

Supplemental Figure 7

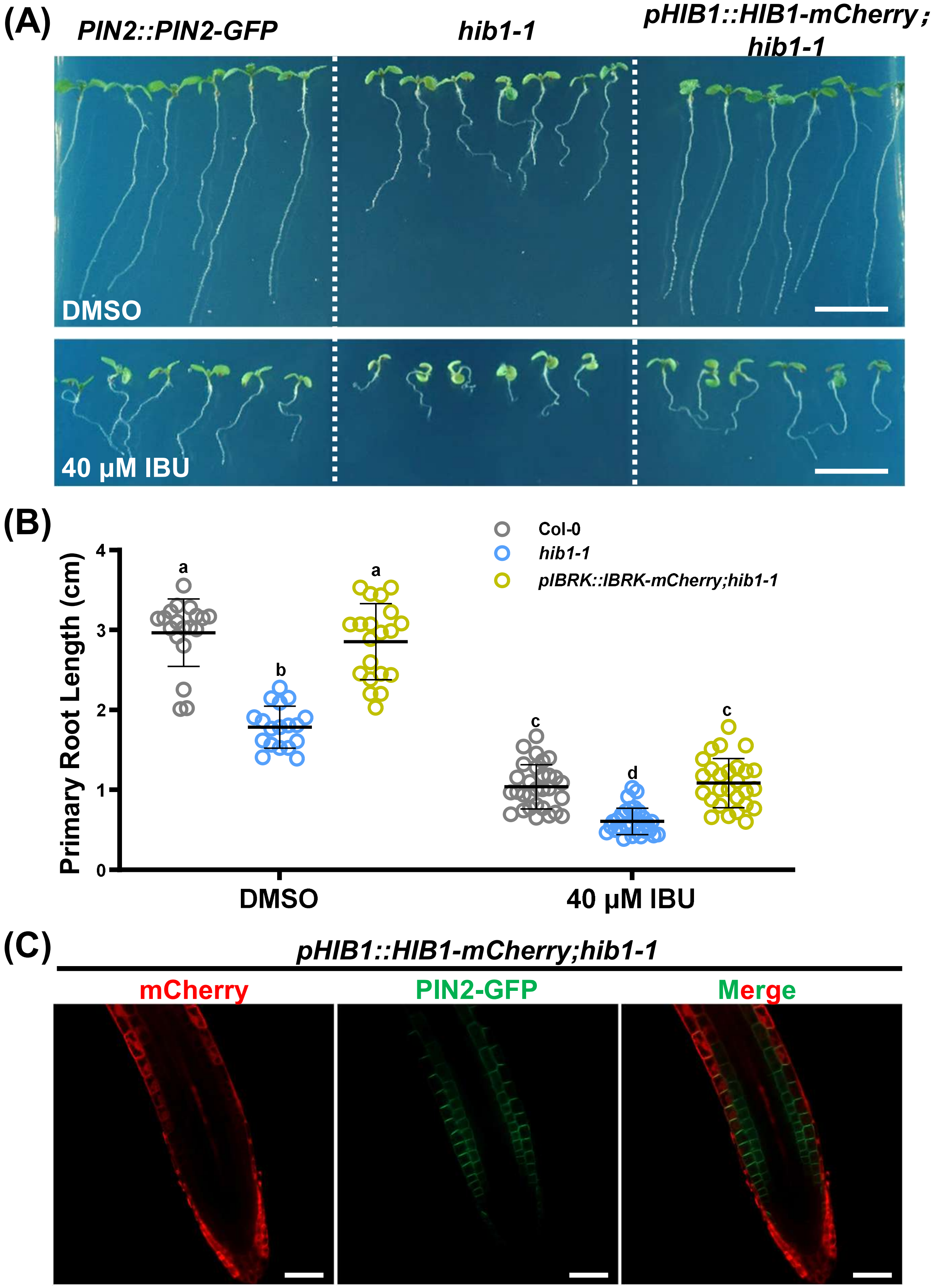

Supplemental Figure 8

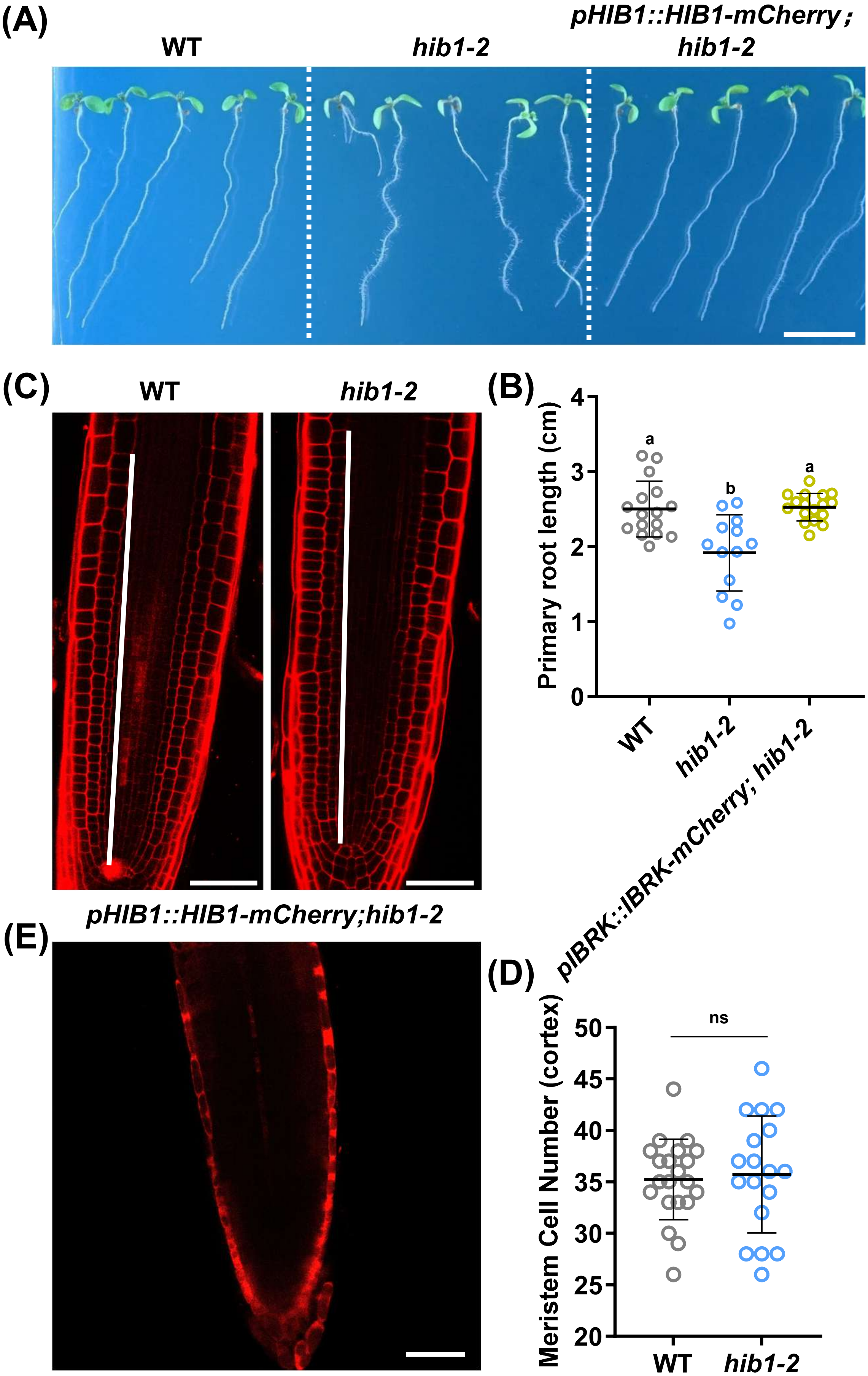

Supplemental Figure 9

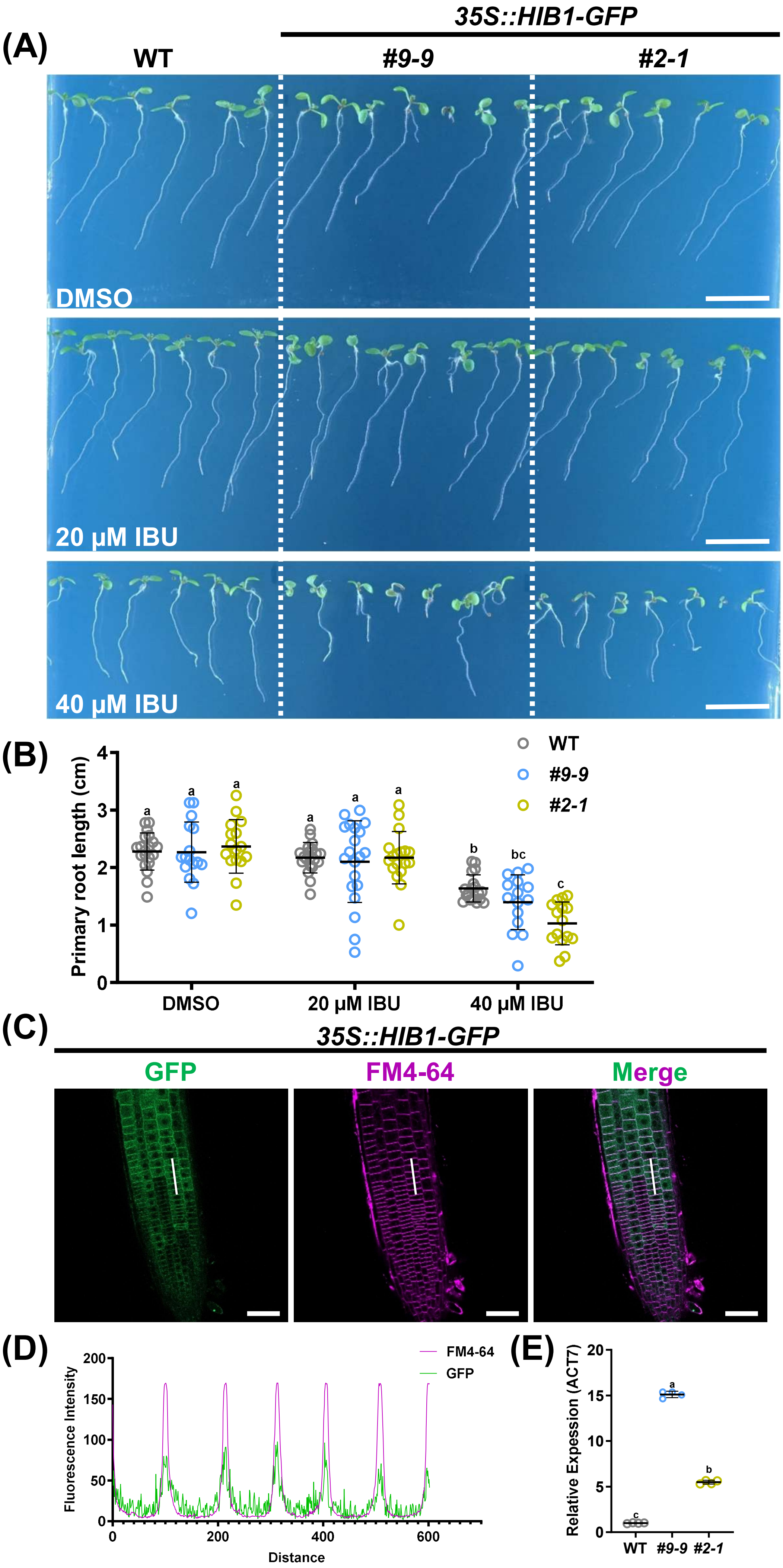

Supplemental Figure 10

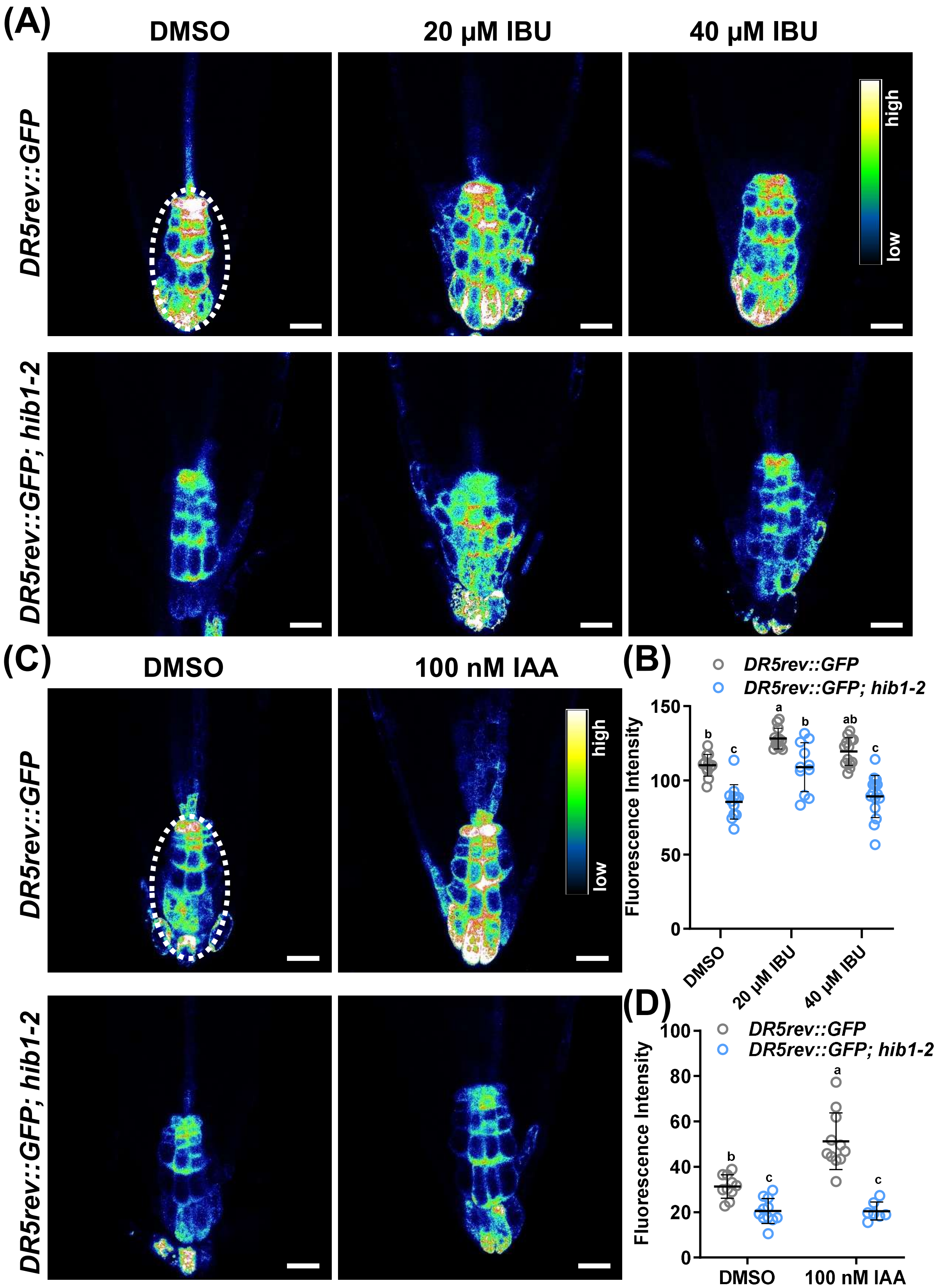

Supplemental Figure 11

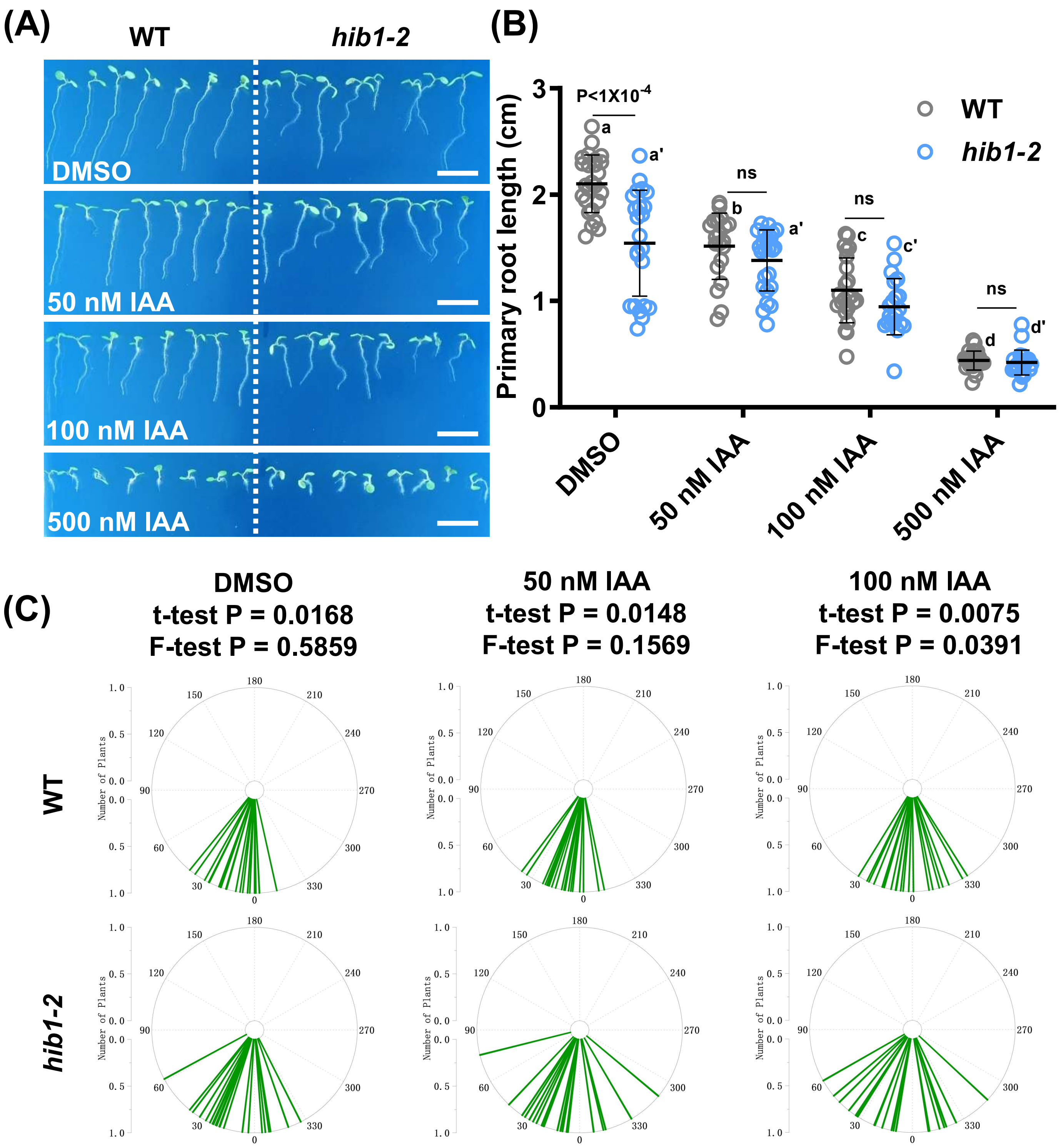

Supplemental Figure 12

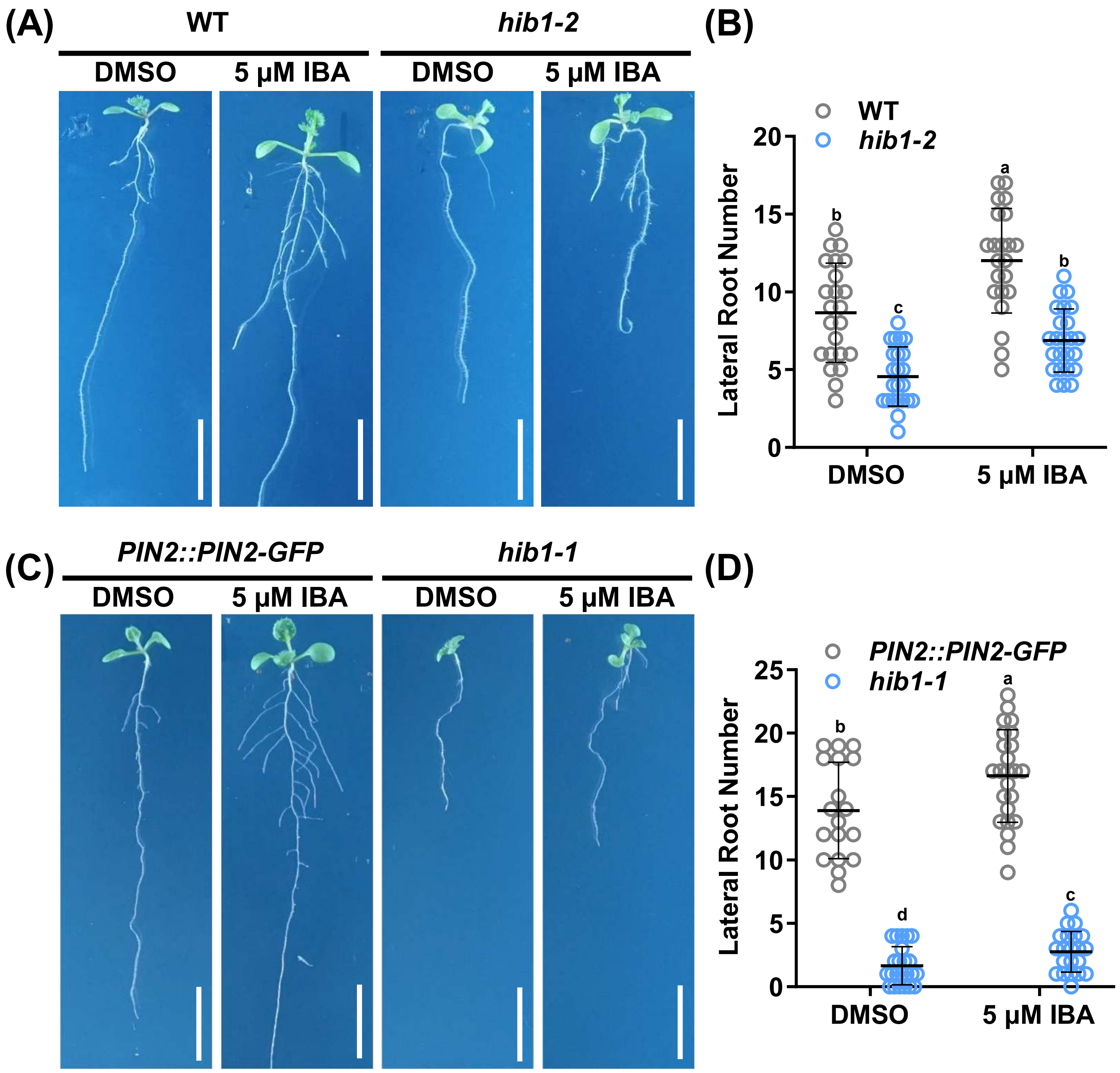

Supplemental Figure 13

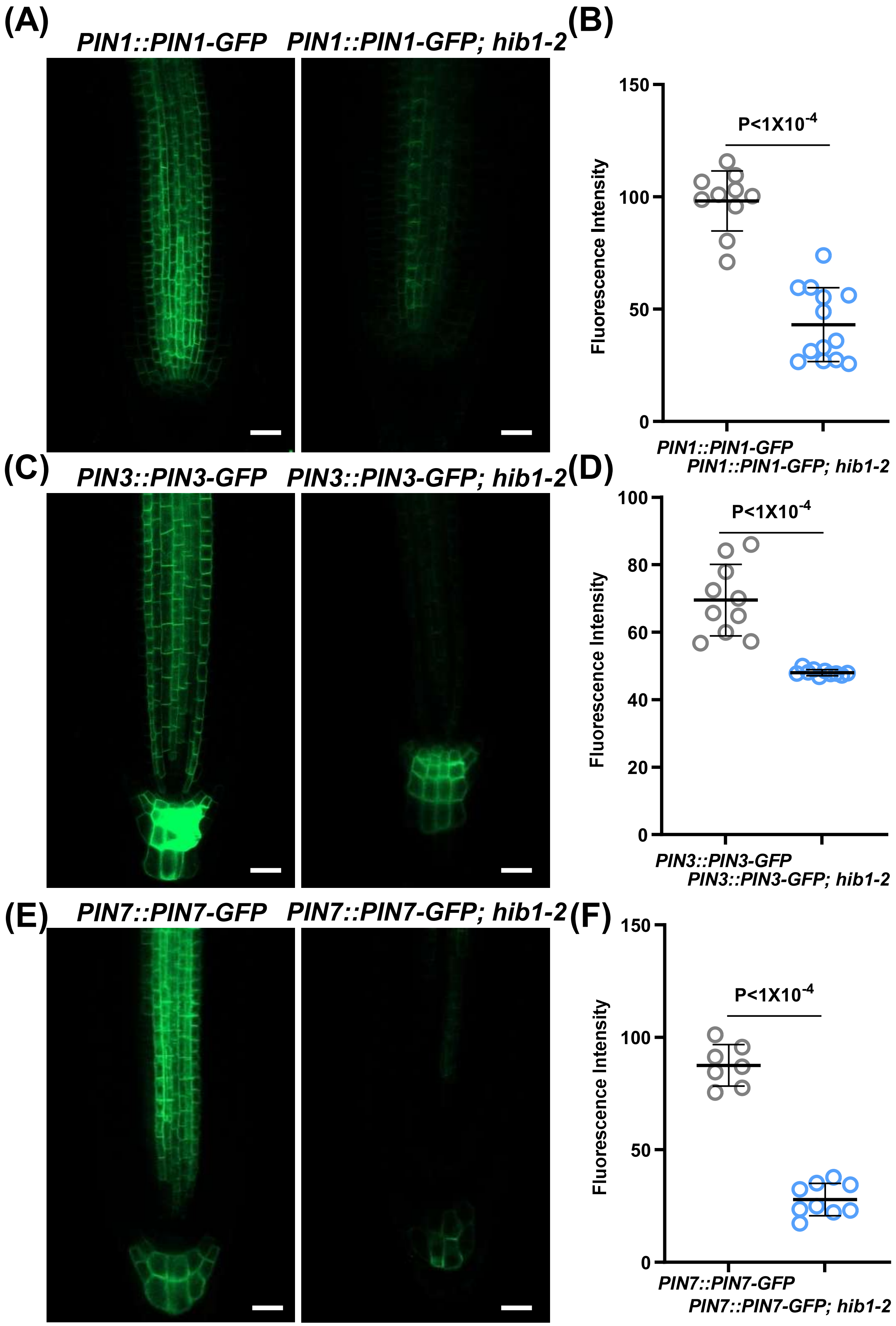

Supplemental Figure 14

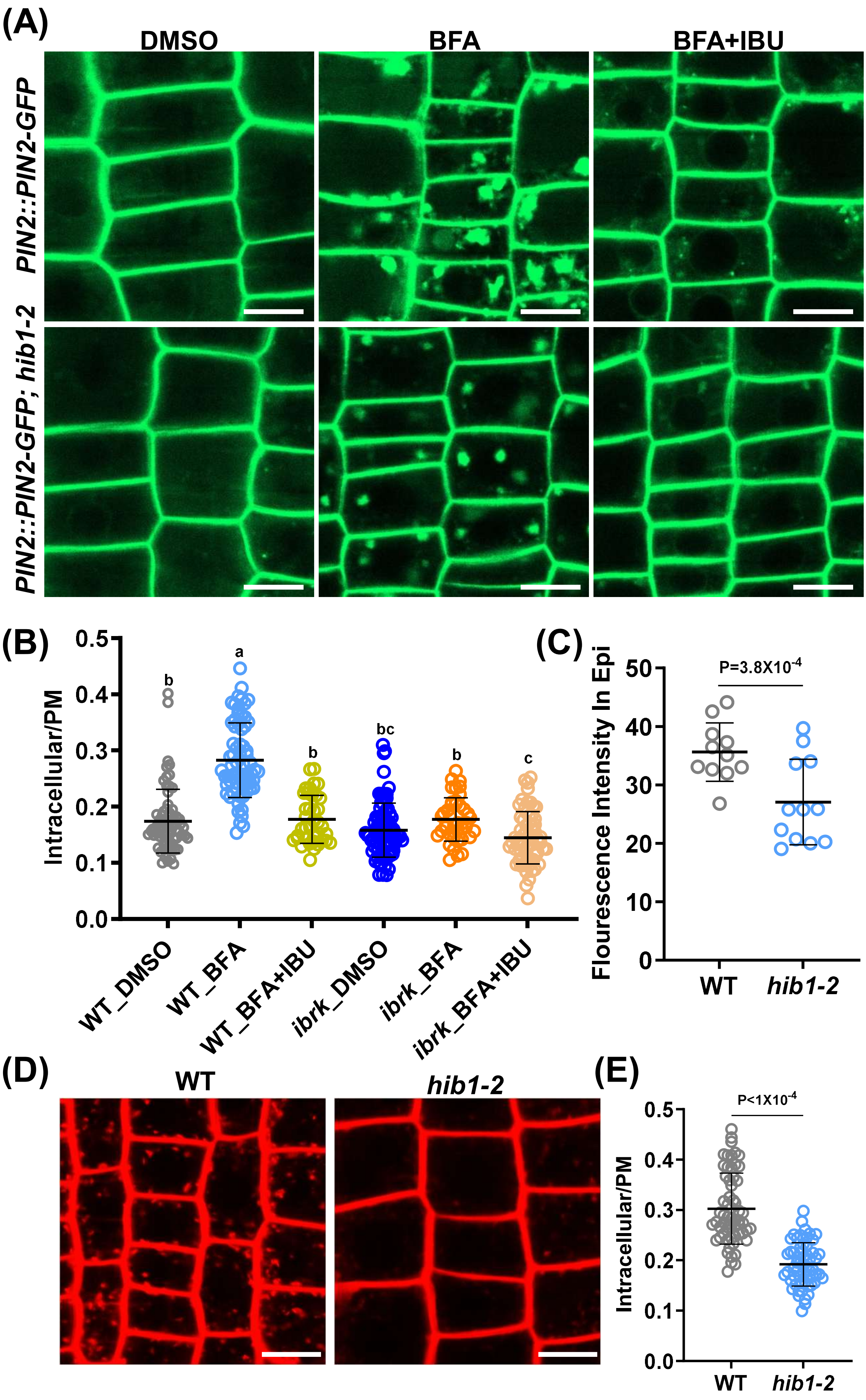

Supplemental Figure 15

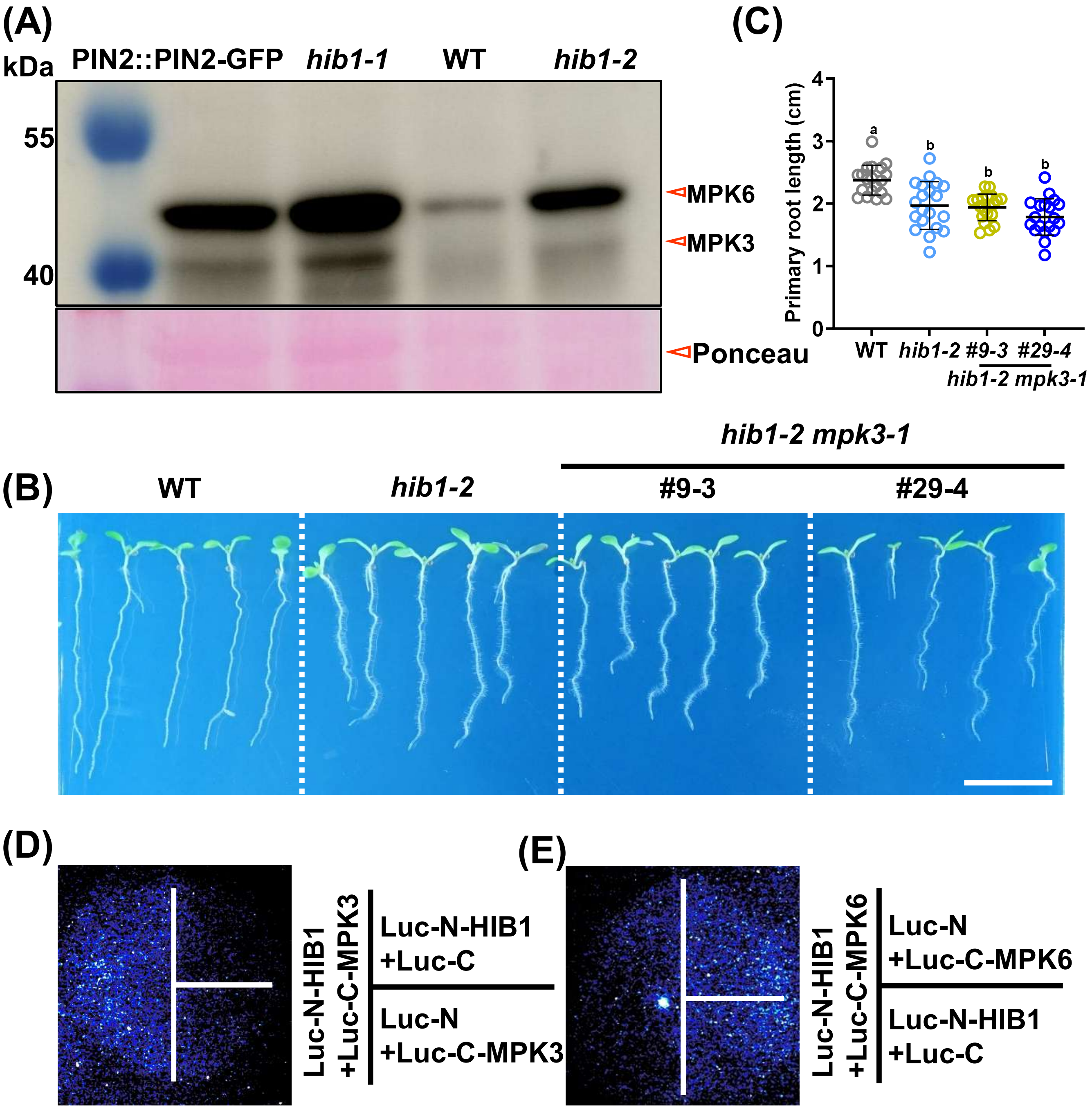

Supplement: Supplementary file 2 [file TPJ-128-0-s002.zip › tpj71206-sup-0001-Figures.pdf]
